# Unraveling the impact of nitric oxide, almitrine, and their combination in COVID-19 (at the edge of sepsis) patients: a systematic review

**DOI:** 10.3389/fphar.2023.1172447

**Published:** 2024-01-22

**Authors:** Ying Wang, Qian Yu, Yuan Tian, Shiying Ren, Liping Liu, Chaojie Wei, Renli Liu, Jing Wang, Dong Li, Kun Zhu

**Affiliations:** ^1^ Department of Pharmacy, China-Japan Union Hospital of Jilin University, Jilin University, Changchun, Jilin, China; ^2^ Department of Immunology, College of Basic Medical Sciences, Jilin University, Changchun, Jilin, China; ^3^ Department of Nutrition and Food Hygiene, School of Public Health, Jilin University, Changchun, Jilin, China; ^4^ Department of Pharmacy, Siping Tumor Hospital, Siping, Jilin, China

**Keywords:** severe acute respiratory syndrome coronavirus 2, coronavirus disease 2019, sepsis, inhaled nitric oxide, almitrine

## Abstract

**Introduction:** During the coronavirus disease 2019 (COVID-19) pandemic, a large number of critically ill and severe COVID-19 patients meet the diagnostic criteria for sepsis and even septic shock. The treatments for COVID-19 patients with sepsis are still very limited. For sepsis, improving ventilation is one of the main treatments. Nitric oxide (NO) and almitrine have been reported to improve oxygenation in patients with “classical” sepsis. Here, we conducted a systematic review and meta-analysis to evaluate the efficacy and safety of NO, almitrine, and the combination of both for COVID-19 (at the edge of sepsis) patients.

**Method:** A systematic search was performed on Embase, PubMed, the Cochrane Library, the Web of Science, Wanfang Data, and China National Knowledge Infrastructure. Randomized clinical trials, cohort studies, cross-sectional studies, case-control studies, case series, and case reports in COVID-19 patients with suspected or confirmed sepsis were performed. Study characteristics, patient demographics, interventions, and outcomes were extracted from eligible articles.

**Results:** A total of 35 studies representing 1,701 patients met eligibility criteria. Inhaled NO did not affect the mortality (OR 0.96, 95% CI 0.33–2.8, I^2^ = 81%, very low certainty), hospital length of stay (SMD 0.62, 95% CI 0.04–1.17, I^2^ = 83%, very low certainty), and intubation needs (OR 0.82, 95% CI 0.34–1.93, I^2^ = 56%, very low certainty) of patients with COVID-19 (at the edge of sepsis). Meanwhile, almitrine did not affect the mortality (OR 0.44, 95% CI 0.17–1.13, low certainty), hospital length of stay (SMD 0.00, 95% CI -0.29–0.29, low certainty), intubation needs (OR 0.94, 95% CI 0.5–1.79, low certainty), and SAEs (OR 1.16, 95% CI 0.63–2.15, low certainty). Compared with pre-administration, the PaO_2_/FiO_2_ of patients with NO (SMD-0.87, 95% CI -1.08–0.66, I^2^ = 0%, very low certainty), almitrine (SMD-0.73, 95% CI-1.06–0.4, I^2^ = 1%, very low certainty), and the combination of both (SMD-0.94, 95% CI-1.71–0.16, I^2^ = 47%, very low certainty) increased significantly.

**Conclusion:** Inhaled NO, almitrine, and the combination of the two drugs improved oxygenation significantly, but did not affect the patients’ mortality, hospitalization duration, and intubation needs. Almitrine did not significantly increase the patients’ SAEs. Well-designed high-quality studies are needed for establishing a stronger quality of evidence.

**Systematic Review Registration:** https://www.crd.york.ac.uk/PROSPERO/display_record.php?RecordID=367667, identifier CRD42022367667.

## 1 Introduction

Sepsis represents a syndrome characterized by pathological, physiological, and biochemical abnormalities instigated by infection (Singer et al., 2016). The clinical manifestations exhibited by a substantial number of critically ill and severely affected patients with coronavirus disease 2019 (COVID-19) meet the diagnostic criteria for sepsis and, in some instances, septic shock. Hypoxemia emerges as a characteristic symptom among individuals with severe COVID-19. The principal mechanism underlying hypoxemia involves the inflammatory-induced pulmonary shunt, along with the loss of surfactant due to alveolar congestion and alveolar collapse (Bersten et al., 1998). Hypoxic pulmonary vasoconstriction (HPV) denotes the inherent mechanism responsible for the automatic regulation of lung oxygen deficiency. In the presence of inadequate oxygen levels within the lung, HPV orchestrates the equilibrium of blood gas ratios, thereby mitigating the incidence of hypoxia. Simultaneously, pulmonary artery pressure serves as a strong negative prognostic indicator in acute respiratory distress syndrome (ARDS) (Squara et al., 1998). Consequently, for patients with severe COVID-19, particularly those with sepsis, augmenting ventilation status assumes paramount significance in addition to interventions such as fluid resuscitation, administration of vasoactive medications, anti-infective therapies, and other treatments.

Inhaled nitric oxide (NO), a specific pulmonary vasodilator initially employed in patients with pulmonary hypertension, exhibits limited systemic activity due to its rapid dissemination into the bloodstream. Consequently, the vasodilatory effects of NO primarily target the pulmonary circulation. By redistributing blood flow to well-ventilated regions, inhaled NO enhances the ventilator–perfusion ratio. In patients with ARDS, the administration of inhaled NO has shown improvements in gas exchange, alleviation of pulmonary hypertension, and mitigation of right ventricular failure (Frostell et al., 1991; Rossaint et al., 1993; Squara et al., 1998). *In vitro* studies have demonstrated that NO donors possess the ability to suppress the replication of certain viruses, including severe acute respiratory syndrome coronavirus 2 (SARS-CoV-2) (Lisi et al., 2021). In addition to these advantageous influences, NO exhibits immunomodulatory and anti-oxidant properties, potentially exerting a constructive impact on COVID-19 (Mir and Maurya, 2021; Mir and Maurya, 2022).

Inflammation has the potential to disrupt the intrinsic mechanism of HPV (Jolin and Bjertnaes, 1991), thereby contributing to ventilation/perfusion (V/Q) mismatch (Sylvester et al., 2012). Consequently, the exploration of selective pulmonary vasoconstrictors has emerged as a consideration. Almitrine, a specific pulmonary vasoconstrictor, has demonstrated the ability to enhance oxygenation in patients with ARDS by augmenting hypoxic pulmonary vasoconstriction (Mélot et al., 1989). Some researchers propose that by reinforcing hypoxic pulmonary vasoconstriction to improve the V/Q ratio, almitrine may attenuate the progression of hypoxemia, potentially obviating the need for mechanical ventilation and reducing the duration of ICU stay and mortality (Kalfon et al., 2022). Furthermore, reports have indicated the use of almitrine in combination with inhaled NO to enhance gas exchange in cases of ARDS, both with and without COVID-19 (Payen et al., 1993). However, NO and almitrine have played a certain role in the treatment of “classical” sepsis, but their efficacy and safety are also controversial.

Therefore, our study was designed to comprehensively assess the effectiveness and safety of almitrine, inhaled NO, and the combined use of inhaled NO in the treatment of patients with sepsis and COVID-19.

## 2 Methods

The systematic review was conducted in accordance with Preferred Reporting Items for Systematic Reviews and Meta-analyses (PRISMA) guideline (Supplementary Appendix S1, appendix p1–8) (Page et al., 2021) and was registered with the National Institute for Health Research international prospective register of systematic reviews (PROSPERO registration number CRD42022367667) (Wang et al., 2022).

### 2.1 Search strategy and selection criteria

Electronic searches were carried out in Embase, PubMed, the Cochrane Library, the Web of Science, Wanfang Data, and China National Knowledge Infrastructure. The search terms we used were “SARS-CoV-2,” “Corona Virus Disease 2019,” “COVID-19,” “nitric oxide,” “NO,” “almitrine,” “iNO,” “NO and almitrine,” “nitric oxide and almitrine,” and relevant keywords for publications until 23.10.2023. The search strategies are available in Supplementary Appendix S1, appendix p9–11. Unpublished and ongoing studies were identified by searching pre-print servers including medRxiv. Searches were carried out by two reviewers (Y.W and K.Z) independently in a standardized manner, followed by screening through titles, abstracts, and full text. Disagreements were resolved by consensus with unresolved conflicts decided by a third reviewer (D.L).

Inclusion criteria were as follows: 1) Patients were confirmed COVID-19 and the SOFA score (absolute, median, and mean value) ≥2, or in accordance with the SOFA scoring tool, a certain system index (absolute, median, and mean value) should be within the scope of corresponding to the system score ≥2, such as the PaO_2_/FiO_2_ ratio (P/F) (absolute, median, and mean value) was less than 300 mmHg (Singer et al., 2016). According to the SpO_2_/FiO_2_ ratio (S/F) = 64 + 0.84*(P/F), the S/F of 315 was approximately equal to a P/F ratio of 300 mmHg (Rice et al., 2007). In this study, we defined that such COVID-19 patients were at the edge of sepsis. 2) The intervention of interest was inhaled NO, intravenous almitrine, or inhaled NO combined with intravenous almitrine with or without standard treatment. Comparator treatments included placebo, standard treatment, and no intervention. No control group studies were included. 3) Randomized clinical trials (RCTs), case-control studies, cohort studies, cross-sectional studies, case reports, case series, and grey literature were included. The language was limited to Chinese and English. Exclusion criteria were as follows: 1) Patients were not confirmed COVID-19. 2) The SOFA score (absolute, median, and mean value) ≤2 or any of the system indicators did not reach 2. 3) Data on SOFA score or certain indicators were not available in the text, Supplementary Materials, or relevant resources. 4) Studies without an available full text or incomplete or unavailable data, conference abstracts, posters, opinion articles, commentaries, animal experiments, and *in vitro* studies. The efficacy outcomes were 28–30 days mortality, in-hospital mortality, P/F, and intubation needs. The safety outcomes were serious adverse events (SAEs) such as acute kidney injury (AKI) (Al Sulaiman et al., 2022).

### 2.2 Search strategy and selection criteria

Two independent reviewers (Y.W and K.Z) extracted the eligible studies, and a third reviewer (D.L) validated them. The extracted information includes the published year, authors, country, study type, sample size, participant demographics, SOFA score, patients’ position, drug dosage, route of administration, control group, mortality outcome, safety outcome, and conclusion of authors.

Included studies were assessed for quality by three reviewers (RL.L, LP.L, and CJ.W) in a standardized process. The Risk of Bias 2.0 tool was used to assess the RCTs (Steudel et al., 1999; Sterne et al., 2019). The methodological quality of case-control and cohort studies was assessed based on the Newcastle–Ottawa Scale (NOS) (NOS, 2020). The methodological quality of the included case reports, case series, and cross-sectional studies was assessed based on JBI critical appraisal tools (JBI, 2020). The reviewers shared the quality assessment results and gained consensus through discussion. The quality of evidence was assessed by using the “Grading of Recommendations Assessment Development and Evaluation (GRADE)” tool (Granholm et al., 2019).

### 2.3 Data synthesis and analysis

The Review Manager v.5.4.1 software was used for statistical analysis. For dichotomous outcomes, the total number of participants and the number of events in each group were recorded. For continuous outcomes, the total number of participants, mean, and standard deviation were recorded. If the authors reported the median and interquartile range, we estimated the mean and standard deviation (Wan et al., 2014; Luo et al., 2018). We report odds ratio (OR) for dichotomous outcomes and standard mean differences (Std MDs) for continuous outcomes. The fixed-effect model was applied when the result of the Q test was not significant (*p* > 0.1) and I^2^<50%. The I^2^ statistic was used to measure heterogeneity (I^2^: 30%–60% was defined as moderate heterogeneity, and 80%–100% was defined as significant heterogeneity). Subgroup analyses would be conducted, if data are appropriate. If we could not implement a meta-analysis, we planned to comment based on the results of included studies.

## 3 Results

### 3.1 Search results

**FIGURE 1:**
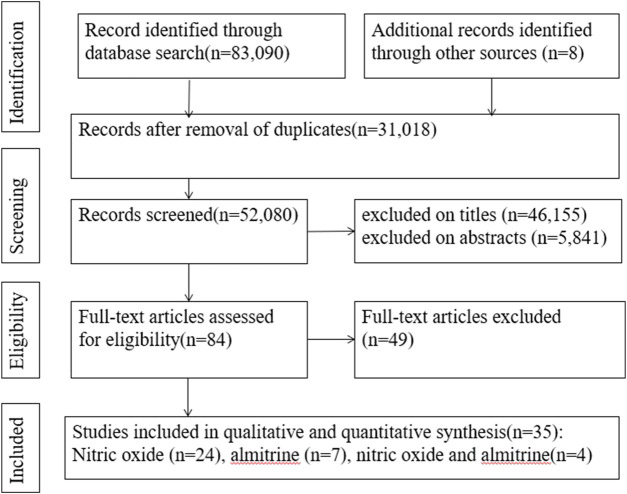
PRISMA flow chart of study selection.

A search of the electronic databases on 23 October 2023 yielded 83,090 studies. After excluding duplicate articles and screening titles and abstracts, 84 articles were evaluated for full-text review. Among these, we found 35 relevant articles (2 RCTs, 19 cohort studies, 1 case-control study, 1 cross-sectional study, and 12 case reports) (Figure 1) (Abou-Arab et al., 2020; Bagate et al., 2020; Barthélémy et al., 2020; Cardinale et al., 2020; Ferrari et al., 2020; Huette et al., 2020; Losser et al., 2020; Safaee Fakhr et al., 2020; Tavazzi et al., 2020; Caplan et al., 2021; Chandel et al., 2021; Feng et al., 2021; Garfield et al., 2021; Giri et al., 2021; Herranz et al., 2021; Heuts et al., 2021; Huette et al., 2021; Laghlam et al., 2021; Longobardo et al., 2021; Lotz et al., 2021; Moni et al., 2021; Paramanathan et al., 2021; Ziehr et al., 2021; Al Sulaiman et al., 2022; Brown et al., 2022; Kalfon et al., 2022; Lubinsky et al., 2022; Poonam et al., 2022; Vives et al., 2022; Bicakcioglu et al., 2023; Blot et al., 2023; Di Fenza et al., 2023; Mekontso Dessap et al., 2023; Saccheri et al., 2023; van Zyl et al., 2023).

### 3.2 Study characteristics

In the 35 studies included, there were a total of 1,701 COVID-19 patients combined with sepsis, of whom 453 received mechanical ventilation. Ten studies reported SOFA scores of enrolled patients, of which three studies reported scores between 2 and 3 (2 for NO and 1 for almitrine), three studies reported scores between 4 and 5 (2 for NO and 1 for almitrine), and seven studies reported a score ≥ 6 (4 for NO and 3 for almitrine). The remaining 21 articles showed patients’ respiratory status, of which 19 studies included patients’ P/F ≤ 150 mmHg (15 for NO, 2 for almitrine, and 2 for NO combined with almitrine) and 2 studies reported patients’ P/F between 150 Hg and 300 mmHg(NO).

Patients usually received standard treatment (or standard of care) based on local guidelines. However, the majority patients were diagnosed with ARDS, and some of them used prone position to improve oxygenation. In addition, there is still no consensus on the dosage of NO and almitrine for such patients. The common dosage of NO is 10–80 parts per million (PPM) up to a maximum of 160–200 PPM, administered by inhalation. In some studies, patients’ dosage was adjusted according to PaO_2_ in arterial blood gas analysis. The usual dosage of almitrine is 2–16 μg/kg/min up to a maximum of 0.5 mg/kg/min, administered by injection. Characteristics of included studies and patients are presented in Table 1.

**TABLE 1:** Demographics of the included studies.

First author	Country, center	Year	Design	Patients	Age (years), mean (SD)	SOFA score or indicators, median (IQR)	Position	Drug	Dosage	Route of administration	Control	Mortality	Safety outcome	Conclusion of authors
Moni, Merlin	India	2021	Phase II open-label, randomized controlled feasibility trial	25; 18M:7F	NO:53.8 (10.1); control: 65.9 (10.8)	SOFA: NO 2.36 (1.34); control 2.73 (2.28)	——	NO	10–80 ppm	Inhalation	Did not receive NO	28-day mortality: NO 0; control 4 (36%)	No adverse effects	Adjuvant inhalation of NO therapy resulted in significant improvements in clinical outcomes and virology
Garfield B	United Kingdom	2021	Cohort	35; 28M:7F	57.65 (8.1)	P/F-mmHg: 102.25 (29.32)	——	NO	20 ppm	Inhalation	——	30-day mortality: 17/35 (48.5%)	——	Inhaled NO may be helpful in COVID-19 patients with refractory hypoxemia
Poonam P. B. H	United States	2022	Retrospective cohort	103; 63M:40F	NO: 57.2 (12.6); control: 62.9 (10.5)	P/F-mmHg: NO 96.8 (124); control 85.1 (28.3)	Prone position: NO 27 (65.9%); control 54 (87.1%)	NO	20–80 ppm	Inhalation	EPO	30-day mortality: NO 29 (70.7%); control 54 (87.1%)	——	No significant difference between inhaled NO and EPO in terms of the duration of mechanical ventilation, change in P/F ratio, ICU, in-hospital mortality in mechanically ventilated patients
Lubinsky A. S	United States	2022	Retrospective cohort	84; 63M:21F	NO: 62 (10.0); control 54 (22.0)	SOFA: NO 8 (4.0); control 9.5 (4.0)	——	NO	10–40 ppm	Inhalation	EPO	30-day mortality was significantly worse in the inhaled EPO group	Adverse events of selective pulmonary dilators were similar	EPO and inhaled NO were not associated with significant improvement in gas exchange in mechanically ventilated patients
Giri, Abhishek R	United States	2021	Cohort	45; 29M; 16F	65.2 (12.2)	SOFA: 4.3 (2.8)	——	NO	5–20 ppm	Inhalation	——	Eight deaths in the pre-intubation group and nine deaths in the post-intubation group	——	Early pre-intubation use of inhaled NO in patients with COVID-19 with hypoxemic respiratory failure may be useful
Vives M	Spain	2022	Case report	1; F	36 years old	P/F: 182 mmHg	Prone positioning	NO	15 ppm	Inhalation	——	Discharged from the ICU	No adverse effects	Inhaled NO could be considered in patients with significant pulmonary arterial hypertension causing right ventricle dysfunction
Ziehr D. R	United States	2021	Retrospective cohort	12	60 (51–71)	P/F-mmHg: 136 (77–168)	Prone positioning	NO		Inhalation	——	——	——	Prone positioning may be a benefit in oxygenation among patients treated with inhaled NO
Heuts S	Netherlands	2021	Case report	1	45 years old	P/F < 80 mmHg	Prone position	NO	20–30 ppm	Inhalation	——	The clinical situation was complicated again	——	Continuous inhaled nitric oxide may be effective in low-tidal volume situations
Brown C. J	United States	2022	Case series	5	42–55 years old	P/F < 80 mmHg	Prone position	NO	20–40 ppm	Inhalation	——	3/5 dead	——	Inhaled NO may improve oxygenation during helicopter transport to a higher care facility
Paramanathan S	Denmark	2021	Case report	1; pregnant	25 years old	P/F: 118 mmHg	Prone position	NO	20 ppm	Inhalation		Discharged		Inhaling NO may be beneficial for pregnant women with COVID-19 after delivery
Lotz C	Germany	2020	Retrospective cohort	7	——	P/F-mmHg: 78.2 (64.5–101.5)	——	NO	20 ppm	Inhalation	——	——	——	Inhaled NO may help and reduce respiratory deterioration in COVID-19-induced ARDS
Al Sulaiman K	Saudi Arabia	2022	Multi-center, retrospective cohort study	210; 120M: 90F	60.1 (14.40)	SOFA: 5.0 (4.00, 8.00)	——	NO	A median dose of 40.0 (32.5, 40.0) PPM	Inhalation	Did not receive NO	30-day mortality: NO group 41 (73.2%); control 44 (36.1%)	Patients with higher odds of AKI and hospital/ventilator-acquired pneumonia after receiving NO	Inhalation of NO has no benefit on mortality for COVID-19 patients with ARDS
Safaee Fakhr B	United States	2020	Cohort	Six pregnant women		P/F < 300 mmHg	——	NO	160–200 ppm	Inhalation		Three patients were discharged. The other three patients’ babies were in good condition	Inhalation of NO was tolerated in pregnant patients	NO is easy to use and safe, which may be beneficial to pregnant women with COVID-19 who suffer from hypoxic respiratory failure
Feng W. X	China	2021	Case reports	3; M	69,65,69	P/F < 100 mmHg	——	NO	10–20 ppm	Inhalation	——	Case 2: died	——	Inhaled NO might reduce the risk of right heart failure in COVID-19 patients with pulmonary hypertension
Abou-Arab O	France	2020	A single-center prospective study	34	——	P/F-mmHg: 70 (63–100)	——	NO	10 ppm	Inhalation	——	ICU mortality: 13	——	If inhaled NO could improve P/F ventilation in severe patients, the reasons of unresponsiveness to NO remain unknown
Ferrari M	Italy	2020	Case series	10	559)	P/F-mmHg: 81 (19)	Prone position	NO	20 ppm	Inhalation	——	Discharged: 8 (80%)	——	NO did not significantly improve arterial oxygenation of COVID-19 with severe hypoxemia
Tavazzi G	Italy	2020	Case series	16; 15M:1F	66.0 (59.6–69.7)	P/F-mmHg: 91.7 (62.1–109.2)	Prone position	NO	25 (20–30) ppm	Inhalation	——	——	——	NO did not improve oxygenation in patients with refractory COVID-19 hypoxemia after prone position
Herranz L	Brazil	2020	Cross-sectional study	27; 19M:8F	60	P/F < 150 mmHg	——	NO	Initial dose: 20–30 ppm. Maximal dose: 40 ppm	Inhalation	Did not receive NO	Mortality was similar in both groups	No major side effect was reported	NO lead to a sustained increase of P/F in mechanical ventilated COVID-19 patients, with no serious side effects
Longobardo A	United Kingdom	2020	A single-center retrospective case-control study	20; 13M:7F	59 (51–65)	P/F: 102 (78–117)	Prone position	NO	20 (10–20) ppm	Inhalation	Non-COVID-19 ARDS	——	——	After NO, the majority of COVID-19 patients with refractory hypoxemia did not show an increase in P/F
Chandel A	United States	2021	Multi-center cohort	272; 180M:92F	57 (13)	SOFA: 3 (1, 5)	——	NO	20–40 ppm	via HFNC	HFNC	Mortality was similar in both groups	No difference was found for AKI.	NO delivered via HFNC did not improve clinical outcomes in patients with COVID-19 respiratory failure
Di Fenza R	United States	2023	Multi-center cohort	193; 128M:65F	62 (IQR 50–70)	SOFA: treatment 8.5 (7–11); control 8 (7–10)	——	NO	80 ppm; 48 h	Inhalation	Did not receive NO	Mortality was similar in both groups	No SAEs were reported	High-dose inhaled NO improves P/F
van Zyl A. G. P	South Africa	2023	Case reports	10	——	P/F:32–71	——	NO	15–20 ppm	Inhalation	——	NO did not reduce mortality	——	NO improves P/F
Bicakcioglu M	Turkey	2023	Case series	16; 11M:5F	——	SOFA: 4–8	Prone position	NO	20 ppm/h	Inhalation	——	NO did not reduce mortality	——	NO improves P/F significantly
Mekontso Dessap A	France	2023	cohort	151; 121M:30F	65 (56–72)	SOFA: 8 (5–12)	Prone position: 68%	NO	10 (7–13) ppm	Inhalation		NO did not reduce mortality	——	NO improves P/F significantly
Barthélémy R	France	2020	Monocenter retrospective study	19; 14M:5F	63 (54–67)	P/F-mmHg: 79 (64–100)	Prone positioning session of at least 16 h	Almitrine	2 μg/kg/min	Intravenous	——	Three deaths	——	After the administration of almitrine (2 μg/kg/min), the patients P/F increased in the following 6 h
Caplan M	France	2021	Single-center retrospective observational study	32; 25M:7F	63 (52–69)	SOFA: 7 (4–10)	Prone positioning: 29 (90.6%)	Almitrine	10 μg/kg/min	Intravenous	——	Responders: 10 (47.6%); non-responders: 7 (63.6%)	Without adverse events	Almitrine infusion improved oxygenation in SARS-CoV-2-induced ARDS
Saccheri C	France	2022	Prospective and observational study	62; 41M:21F	Non-responders: 67 [56–71]; responders: 62 [53–67]	SOFA: 2 (2–3)	Prone positioning was stopped early	Almitrine	16 μg/kg/min over 30 min	Intravenous	——	Responders: 4 (9%); non-responders: 0	No patients experienced hemodynamic adverse effects	Almitrine could affect oxygenation
Kalfon P	France	2022	Randomized, double-blind, placebo-controlled, multicentre trial	179; 119M:60F	Almitrine group: 59.9 (11.6); placebo group: 60.3 (11.9)	SOFA: 4.0 (3.0–4.0)	Prone positioning: almitrine group 46 (53%); control group 54 (60%)	Almitrine	2 μg/kg/min	Intravenous	Placebo	28-day mortality: almitrine group 7 (8%); placebo group 15 (16%)	The adverse reactions in the two groups were similar, both slight	Almitrine (2 μg/kg/min) did not reduce the death at day 7
Losser M. R	France	2020	Case series	10; 10 M	70 (54–78)	P/F-mmHg: 135 (85, 195)	Prone positioning: 3 (30%)	Almitrine	4 or 12 μg/kg/min	Intravenous	——	Four patients (24%) died in the ICU.	——	For early COVID-19 with severe hypoxemia, almitrine infusion is associated with improved oxygenation
Huette P	France	2021	Case reports	3; 1M:2F	53, 56, 57 years old	SOFA: 7, 8, 13	Supine position	Almitrine	4 μg/kg/min	Intravenous	——	Discharged from the ICU	NO adverse effects on the right ventricular function	Almitrine may be helpful in enhancing oxygenation in COVID-19 patients
Blot P. L	France	2023	Cohort	60; 42M:18F	64 [54–70]	SOFA: 7 (4–11]	Prone positioning: (76%)	Almitrine	2 μg/kg/min	Intravenous	Non-COVID-19 ARDS	P/F increased after almitrine infusion	——	After almitrine infusion, the increase in P/F was higher in non C-19-ARDS than in C-ARDS
Huette P	France	2020	Case report	1F	57	P/F: 70 mmHg	Prone positioning	NO and almitrine	10 ppm; 4 μg/kg/min	Inhalation; intravenous	——	Discharged	Almitrine-related reversible lactic acidosis and hepatic dysfunction were not observed	Almitrine infusion improved oxygenation and right ventricle function
Laghlam D	France	2021	Observational, single-center, open-label study	12; 9M:3F	71.8 (8.7)	P/F-mmHg: 146 (48)	At least one session of ventilation with prone position	NO and almitrine	10 ppm; 8 μg/kg/min	Inhalation; central venous	——	90-day mortality: 50%	No adverse event was observed	Combining almitrine and NO improved the short-term oxygenation
Cardinale M	France	2020	Retrospective study	20	73 (45–76)	P/F: 88 (73–110) mmHg	——	NO and almitrine	10–20 ppm; 0.5 mg/kg/min	Inhalation; intravenous	——	——	——	In the moderate-to-severe ARDS induced by COVID-19, the use of NO or almitrine, or a combination of both, did not improve oxygenation
Bagate F	France	2020	Cohort	10; 7M:3F	60 (52–72)	SOFA: 6 (3–7)	Prone position lasting 16–18 h	NO and almitrine	NO: 10 ppm; Almitrine: 10 µg//kg/min	Inhalation; intravenous	Inhaled NO	——	No side event was observed	NO combined with almitrine was associated with a significant and rapid improvement of oxygenation

PEEP, positive end-expiratory pressure; Pplat, plateau pressure; P/F, PaO_2_/FiO_2_; ARDS, acute respiratory distress syndrome; ICU, intensive care unit; NO, nitric oxide; EPO, epoprostenol; PPM, parts per million; AKI, acute kidney injury; HFNC, high-flow nasal cannula.

### 3.3 Assessment of study quality

The risk of bias of two RCTs was low to moderate (Supplementary Appendix S2, appendix p1–2). The methodological quality of 18 cohorts were moderate to high, and one case-control study was moderate (NOS assessment results are shown in Supplementary Appendix S2, appendix p3–22). The methodological quality of four case series was moderate, eight case reports was moderate to high, and one cross-sectional study was moderate (JBI assessment results are shown in Supplementary Appendix S2, appendix p23–35). Supplementary Appendix S2 (appendix p38–41) summarized the result of GRADE assessment for the certainty of evidence.

### 3.4 Results of meta-analysis

#### 3.4.1 Mortality outcomes

**FIGURE 2:**
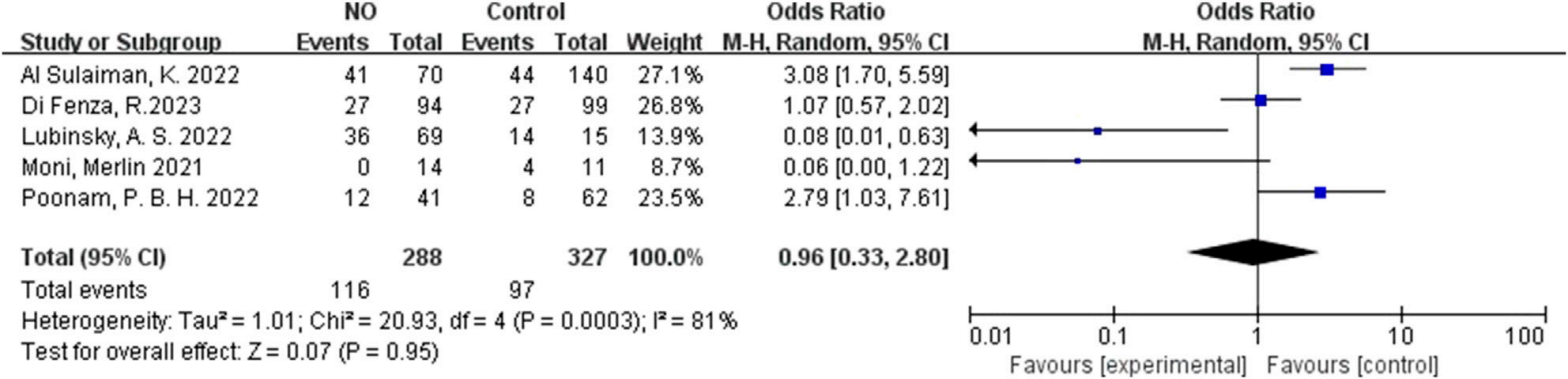
Mortality at 28–30 days of inhalation NO.

**FIGURE 3:**
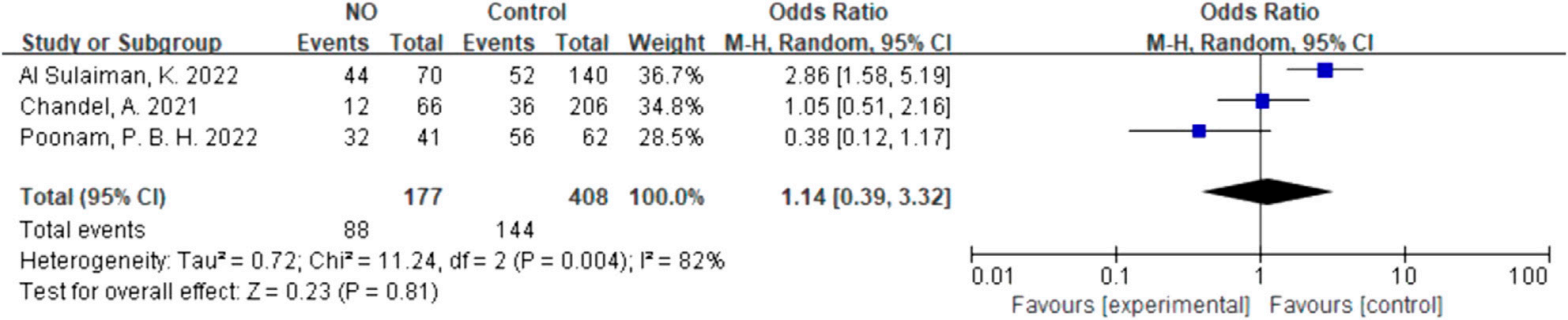
In-hospital mortality of inhalation NO.

**FIGURE 4:**
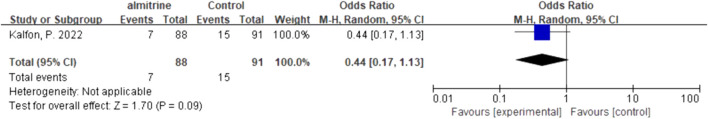
Mortality at 28–30 days of almitrine.

**FIGURE 5:**
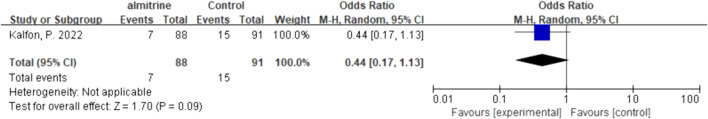
In-hospital mortality of almitrine.

For inhalation NO, one RCT and four cohorts reported mortality at 28–30 days, and three cohorts reported hospital mortality. For almitrine, only one RCT reported mortality at 28–30 days and hospital mortality. Compared to the control group, inhaled NO might decrease mortality at 28–30 days (OR 0.96, 95% CI 0.33–2.8, I^2^ = 81%), but there was no significant difference between the two groups (Figure 2). In addition, inhaled NO might increase hospital mortality (OR 1.14, 95% CI 0.39–3.32, I^2^ = 82%), but there was no significant difference between the two groups (Figure 3). Compared to the control group, almitrine might decrease mortality at 28–30 days (OR 0.44, 95% CI 0.17–1.13), but there was no significant difference between the two groups (Figure 4). The result was similar to hospital mortality (OR 0.44, 95% CI 0.17–1.13) (Figure 5).

#### 3.4.2 Synthesized data of P/F before and after administration

Due to the lack of inter-group data, we analyzed P/F before and after administration. Compared with pre-administration, the P/F of patients after the use of NO, almitrine, and NO–almitrine combination increased significantly (Table 2; Supplementary Appendix S2, Supplementary Figures S3–S5, appendix p36).

**TABLE 2:** Synthesized data of P/F before and after administration.

Medication	Std. MD	95% CI
NO	−0.87	−1.08, −0.66
Almitrine	−0.73	−1.06, −0.40
NO combined with almitrine	−0.94	−1.71, −0.16

#### 3.4.3 Hospital length of stay

**FIGURE 6:**
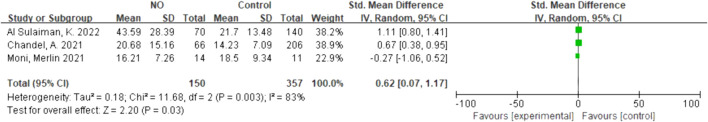
Hospital length of stay of inhalation NO.

**FIGURE 7:**
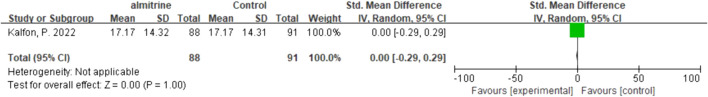
Hospital length of stay of almitrine.

Due to the lack of data on the NO-almitrine combination, we used quantitative synthesis of the hospital length of stay of the patients treated with NO and almitrine alone. Compared to the control group, inhaled NO might shorten the hospital length of stay (SMD 0.62, 95% CI 0.07–1.17, I^2^ = 83%), but there was no significant difference between the two groups (Figure 6). For using almitrine, there was no difference in hospital length of stay between the intervention group and control group (SMD 0.00, 95% CI -0.29–0.29) (Figure 7).

#### 3.4.4 Needs for intubation

**FIGURE 8:**
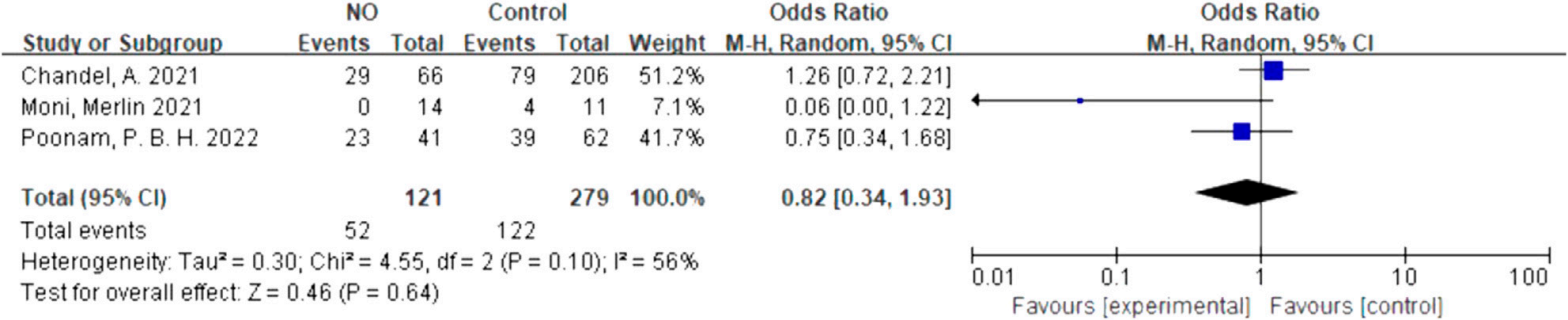
Need for intubation of inhalation NO.

**FIGURE 9:**
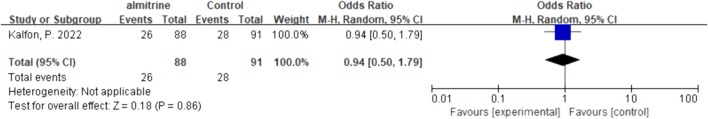
Need for intubation of almitrine.

We quantitatively synthesized the intubation needs of the patients treated with NO and almitrine alone, due to a lack of data on NO combined with almitrine. For inhalation of NO, one RCT and two cohorts reported the need for intubation. For almitrine, only one RCT reported this outcome. Compared to the control group, inhaled NO might reduce the need for intubation, but there was no significant difference between the two groups (OR 0.82, 95% CI 0.34–1.93, I^2^ = 56%) (Figure 8). The RCT of almitrine showed similar trend (OR 0.94, 95% CI 0.5–1.79) (Figure 9).

#### 3.4.5 Safety outcomes

**FIGURE 10:**
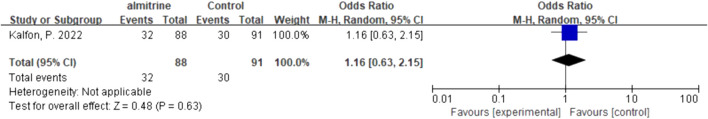
SAEs of almitrine.

Due to significant heterogeneity (Supplementary Appendix S2, Supplementary Figure S6, appendix p37) and a lack of studies on safety outcomes, we only reported the result of quantitative synthesis for almitrine alone. Compared to the control group, almitrine might increase the SAEs, but there was no significant difference between the two groups (OR 1.16, 95% CI 0.63–2.15) (Figure 10).

## 4 Discussion

Critically ill and severe patients admitted to the ICU due to COVID-19 are at a higher risk of progressing to viral sepsis, and these patients would face more complex treatment. Data have shown that sepsis is one of the causes of death in patients with COVID-19 worldwide (Karakike et al., 2021). Among the many vulnerable organs in sepsis patients, the lung is the most vulnerable target organ, and patients often develop ARDS early, which is also one of the causes of death in sepsis patients (Tang and Tan, 2017). Therefore, sepsis and ARDS are not completely separate diseases in clinical treatment. At present, respiratory support is still the main treatment for sepsis and ARDS (Li et al., 2021), with active removal of pathogens and symptomatic support. In addition to ARDS, sepsis may also show other organ dysfunction in the clinic, such as coagulation function, liver and kidney function, or central nervous system dysfunction. Overall, whether sepsis, ARDS, or COVID-19, there is an urgent need for more effective drugs. Unfortunately, medications for sepsis are limited and their efficacy and safety are controversial. Despite significant clinical and basic research efforts in sepsis (especially virus-associated sepsis), there are few effective drugs for this disease worldwide, and no definitive treatment recommendations have been made in authoritative guidelines (Evans et al., 2021). Considering the urgency of sepsis treatment during the COVID-19 pandemic, people are trying to screen out drugs with a potential therapeutic value from previous treatments.

Higher incidences of pulmonary microthrombus and significant vascular endothelial injury were observed in critically ill patients with COVID-19 (Prakash et al., 2021), leading to poor oxygenation and pulmonary changes (Marini and Gattinoni, 2020). The vast majority of critically ill patients require mechanical ventilation owing to difficulties in maintaining oxygenation and ventilation, which remains a major challenge for critically ill patients of COVID-19 (Prakash et al., 2021). During the COVID-19 pandemic, it is essential to increase the number of days without ventilators for critically ill patients and minimize the need for respiratory support equipment. Therefore, dilating smooth muscle vessels and increasing alveolar blood flow to enhance oxygenation may be an option for treating critical illness (Longobardo et al., 2021). NO induces the relaxation of vascular smooth muscle and dilates pulmonary blood vessels, thereby increasing blood oxygenation and reducing the right-to-left shunt in the lung (Yu et al., 2019). Almitrine reduces intrapulmonary shunt by enhancing hypoxic pulmonary vasoconstriction (Reyes et al., 1988; Payen et al., 1993; Wysocki et al., 1994), which has been used for severe hypoxemia patients (Ranieri et al., 2012). NO and almitrine have been reported as a rescue strategy for “classical” ARDS in patients with severe hypoxemia. This treatment increased the P/F and reduced physiologic dead space fraction over 24 h (Reyes et al., 1988; Gebistorf et al., 2016). Almitrine and inhalation of NO were considered by some experts as a salvage treatment strategy for critically ill patients during the COVID-19 pandemic (Spieth and Zhang, 2014; Blot et al., 2023), including refractory hypoxemia. However, these treatments remain controversy. In this study, we found that NO, almitrine alone, and the combination of both significantly improved oxygenation in patients with COVID-19 (at the edge of sepsis), but did not affect the mortality, length of stay, or intubation needs of patients. In the face of the new medical challenge of COVID-19-induced sepsis, further research is still needed. In addition, the specific population and the specific circumstances in which these drugs are needed are to be studied and explored.

### 4.1 The combination of almitrine and NO

NO is a potent pulmonary vasodilator, while almitrine constricts pulmonary blood vessels. The combination of NO and almitrine appears to be contradictory, but some experts have utilized this combination as a rescue measure for critically ill patients (Laghlam et al., 2021). The rationale behind this combination lies in its potential to enhance the ventilation/perfusion ratio (V/Q) through selective vasoconstriction of pulmonary vessels in non-ventilated areas and selective vasodilation of pulmonary vessels in ventilated areas. Our findings indicate that this combination did not improve patient survival but did enhance oxygenation. It is crucial to exercise caution when employing this combination until further clarification regarding the mechanism, timing, and dosage is obtained. Additionally, almitrine is frequently used in conjunction with another drug called raubasine, which acts as a vasodilator, for the treatment of age-related cerebral disorders (Allain and Bentué-Ferrer, 1998) and certain pulmonary diseases. Notably, raubasine has demonstrated anti-SARS-CoV-2 effects in *in vitro* and animal studies, suggesting its therapeutic potential (Kumar et al., 2021; Mohseni et al., 2022).

### 4.2 Pregnant women

Middle East respiratory syndrome (MERS) and severe acute respiratory syndrome coronavirus (SARS) have caused a large number of infectious deaths in pregnant women over the past 20 years. Because pregnant women have elevated levels of progesterone and estrogen, restricted lung expansion is more susceptible to pathogens (Selim et al., 2020). At present, there is still a lack of targeted respiratory interventions for pregnant women with hypoxic respiratory failure due to COVID-19 pneumonia, other than supplemental oxygen and mechanical ventilation. In another study of six pregnant women with severe or critical forms of COVID-19, 160–200 ppm of NO was found to be applied frequently, which appears to be well tolerated and may be beneficial to the people with hypoxic respiratory failure (Safaee Fakhr et al., 2020). Studies have shown that in patients with COVID-19, this innovative breathing intervention is feasible in pregnant women. At present, there have been many studies on the application of NO in the treatment of sepsis (non-COVID-19), but the experience of applying NO in the treatment of pregnant patients with COVID-19 remains very valuable.

### 4.3 Responders and non-responders

Most studies defined the responders as P/F increase >20% or 10 mmHg after administration (Ichinose et al., 2004; Charron et al., 2011). Whether patients with COVID-19 or without COVID-19 were “responders” to NO and almitrine and elements that predict potential responsiveness remain unclear. Manktelow C. et al. reported about 30%–40% of non-COVID-19 ARDS patients were non-respondence to inhaled NO to 67% of the patients with septic shock. They observed that septic shock was a significant predictor of NO inhalation responsiveness (Manktelow et al., 1997). Trachsel S. et al. reported endotoxin-exposed pigs that received inhaled NO responded by producing more endothelin-1, however, with higher levels in the responder group compared to the non-responder group (Trachsel et al., 2008).

As for COVID-19, among the included literature, only three studies compared the baseline characteristics of responders and non-responders, and the oxygenation of responders was lower than that of non-responders (Abou-Arab et al., 2020; Caplan et al., 2021; Garfield et al., 2021). Garfield B reported responders to inhaled NO also had higher baseline brain natriuretic peptides (Garfield et al., 2021). In addition, the prognosis of responders and non-responders is uncertain. Abou-Arab O. et al. stated that responders had a lower 28-day mortality rate (Caplan et al., 2021), and one study reported the ICU mortality of responders was similar to that of non-responders (*p* = 1.0) (Abou-Arab et al., 2020). During the pandemic, more in-depth research is needed on NO and almitrine responsiveness.

### 4.4 COVID-19-associated complications and NO

Infection by SARS-CoV-2 elicits a spectrum of complications, encompassing ARDS, AKI, and myocardial injury (Oxley et al., 2020; Ye et al., 2020). NO exerts selective dilation of pulmonary vessels within ventilated lung units, thereby improving ventilation/perfusion matching while averting systemic hypotension. Consequently, NO has been investigated as a potential treatment for COVID-19-associated ARDS. Moreover, NO may possess cardioprotective properties, as it can attenuate subclinical myocardial injuries normally observed during cardiopulmonary bypass procedures (Redaelli et al., 2022). Hence, this offers novel therapeutic avenues for managing COVID-19-related myocardial damage. Furthermore, inhaled NO could potentially yield favorable hemodynamic effects during cardiopulmonary bypass, thereby enhancing cardiac output and subsequently improving renal perfusion (Rezoagli et al., 2017). Utilizing NO may, thus, confer beneficial effects on cardiac output and provide renal function protection in patients with COVID-19 complicated by cardiovascular disorders.

### 4.5 Safety outcomes

A non-COVID-19 study showed that the plasma concentration and efficacy of almitrine increased in a dose-dependent manner, and perhaps its adverse events seemed to be also dose-dependent (Gallart et al., 1998). In this study, we found that adverse effects of 2 μg/kg/min almitrine were mild and infrequent, and the incidence was similar to that of the placebo groups (Kalfon et al., 2022). Meanwhile, no significant adverse events were observed by the investigators after the use of almitrine at a dose of 4-12 μg/kg/min (Losser et al., 2020; Caplan et al., 2021; Laghlam et al., 2021). However, considering the increase in the incidence rate of pulmonary thromboembolism in COVID-19 patients (Michard et al., 2001), some researchers recommended that almitrine should be used with caution, and the right ventricular loading conditions should be paid attention to after administration (Poissy et al., 2020).

A systematic review reported that inhaled NO might increase the risk of renal dysfunction, especially in patients with prolonged use and ARDS (non-COVID-19 (Ruan et al., 2015)). A multi-center cohort study included in this study showed that moderate-to-severe ARDS in critically ill patients of COVID-19 who received inhaled NO illustrated significantly higher odds of AKI (Al Sulaiman et al., 2022). A latest study on SAEs of NO showed that inhaled NO was associated with severe AKI and renal replacement therapy in critically ill patients of COVID-19 (Bobot et al., 2022). During the pandemic, some researchers tried to use NO for pregnant women with severe to critical COVID-19, and no acute adverse events related to NO were observed (Safaee Fakhr et al., 2020; Valsecchi et al., 2022). Based on the limited evidence, we suggest that doctors balance the benefit-to-risk ratio before prescribing NO for patients with COVID-19 (at the edge of sepsis) and pay attention to the renal function during administration.

### 4.6 Limitation

First, some results of this study have significant heterogeneity, but due to the lack of research, subgroup analysis and meta-regression cannot be carried out. However, despite the high heterogeneity of the research results, they still reflect the trend of the efficacy of NO and almitrine. Meanwhile, the SOFA score or related indicators of included patients were mean or median, so we speculated that not all patients confirmed sepsis, but the results of the patients still reflected a trend because some of these patients might or would develop sepsis. Second, a lack of high-quality clinical research studies limited our analyses. The majority of included studies were retrospective studies; these aspects could have introduced various confounders given the lack of risk adjustment or propensity score weighting. We included studies written only in English and Chinese, which also limits the scope of the review. Third, we found that the SOFA scores of included patients varied, and factors such as ethnic differences, the use of vasoactive drugs in many patients, and prone position had uncertain effects. Few studies analyzed the impact of these factors further and drew conclusions. Both ARDS and sepsis showed individual differences, which may also increase heterogeneity. In addition, the doses of NO and almitrine in the included studies were not uniform, and differences in management schemes for patients with sepsis in different countries and additional variability during the COVID-19 pandemic would increase the heterogeneity of the findings.

## 5 Conclusion

This systematic review demonstrated that both use of NO and almitrine alone, and the combination of the two drugs, could significantly improve oxygenation in patients. NO and almitrine might reduce the mortality, hospital length of stay, and intubation needs of patients, but there is no statistical significance, and almitrine did not significantly affect the SAEs. However, given the lack of clinical data, this conclusion needs more high-quality clinical evidence to verify. Moreover, there is no consensus on the dosage, applicable population, and respondent prediction of these drugs until now, which also increases the uncertainty of the conclusion of this study.

## Data Availability

The original contributions presented in the study are included in the article/Supplementary Material; further inquiries can be directed to the corresponding authors.
