# Supplementary material for: Unraveling the impact of nitric oxide, almitrine, and their combination in COVID-19 (at the edge of sepsis) patients: a systematic review
Source: Front Pharmacol. 2024 Jan 22;14:1172447. doi: 10.3389/fphar.2023.1172447 (PMC10839063; doi:10.3389/fphar.2023.1172447)
Supplement: Supplementary file 1 [file DataSheet2.docx]

**Supplementary 2**

| Supplementary 2 appendix | Page |
| --- | --- |
| Supplementary 2 figure S1-S2. Risk of bias of RCTs | **1-2** |
| Supplementary 2 table 1. NOS assessment results of cohort studies | **3-21** |
| Supplementary 2 table 2.NOS assessment results of case control studies | **22** |
| Supplementary 2 table 3. JBI assessment results of case reports | **23-30** |
| Supplementary 2 table 4. JBI assessment results of case series | **31-34** |
| Supplementary 2 table 5. JBI assessment results of cross-sectional study | **35** |
| Supplementary 2 figure S3-S6. | **36-37** |
| Supplementary 2 table 6. Grade of evidence | **38-41** |

**Supplementary 2 figure S1-S2. Risk of bias of RCTs**


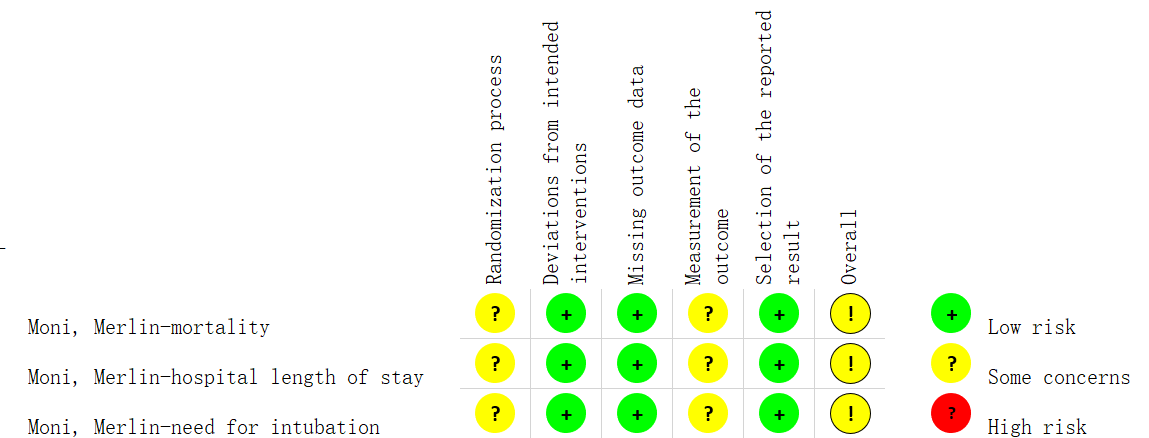


**Figure S1 shows the quality evaluation results of RCT of nitric oxide.**


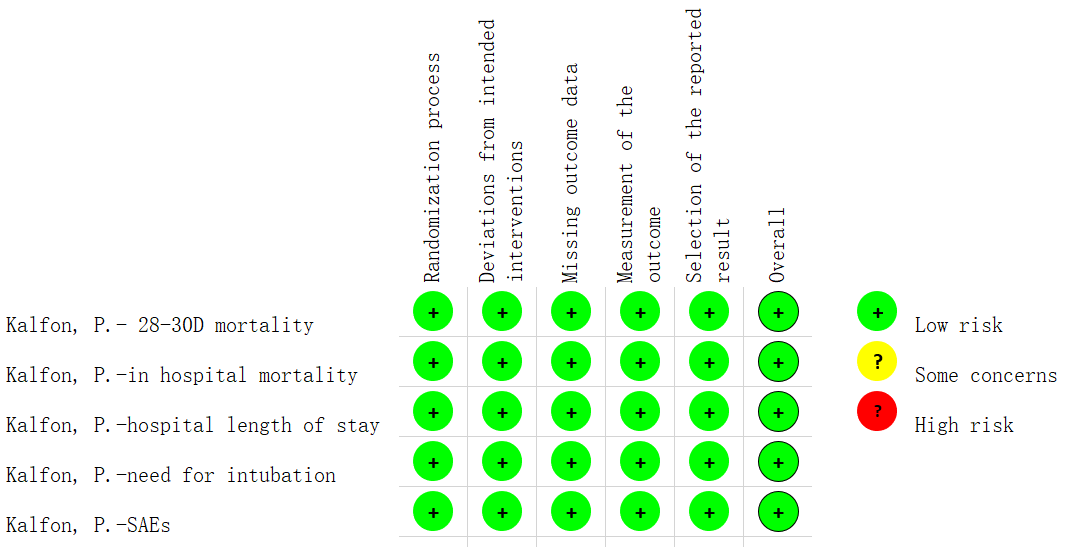


**Figure S2 shows the quality evaluation results of RCT of almitrine.**

**Supplementary 2 table** **1. NOS assessment results of cohort studies**

| **Study name:** Laghlam, D.****** | |
| --- | --- |
| **Items** | **Response options** |
| Selection | |
| *Representativeness of the exposed cohort？* | *** |
| *Selection of the non exposed cohort* |  |
| *Ascertainment of exposure* | *** |
| *Demonstration that outcome of interest was not present at start of study* | *** |
| Comparability | |
| *Comparability of cohorts on the basis of the design or analysis* |  |
| Outcome | |
| *Assessment of outcome* | *** |
| *Was follow-up long enough for outcomes to occur* | *** |
| *Adequacy of follow up of cohorts* |  |

Studies with scores of 0-3, 4-6, 7-9 were, respectively, considered as low, moderate, and high quality.

Moderate quality

| **Study name:** Garfield, B.****** | |
| --- | --- |
| **Items** | **Response options** |
| Selection | |
| *Representativeness of the exposed cohort？* | *** |
| *Selection of the non exposed cohort* |  |
| *Ascertainment of exposure* | *** |
| *Demonstration that outcome of interest was not present at start of study* | *** |
| Comparability | |
| *Comparability of cohorts on the basis of the design or analysis* |  |
| Outcome | |
| *Assessment of outcome* | *** |
| *Was follow-up long enough for outcomes to occur* | *** |
| *Adequacy of follow up of cohorts* |  |

Studies with scores of 0-3, 4-6, 7-9 were, respectively, considered as low, moderate, and high quality.

Moderate quality

| **Study name:** Poonam, P. B. H.****** | |
| --- | --- |
| **Items** | **Response options** |
| Selection | |
| *Representativeness of the exposed cohort？* | *** |
| *Selection of the non exposed cohort* | *** |
| *Ascertainment of exposure* | *** |
| *Demonstration that outcome of interest was not present at start of study* | *** |
| Comparability | |
| *Comparability of cohorts on the basis of the design or analysis* | *** |
| Outcome | |
| *Assessment of outcome* | *** |
| *Was follow-up long enough for outcomes to occur* | *** |
| *Adequacy of follow up of cohorts* |  |

Studies with scores of 0-3, 4-6, 7-9 were, respectively, considered as low, moderate, and high quality.

High quality

| **Study name:** Lubinsky, A. S.****** | |
| --- | --- |
| **Items** | **Response options** |
| Selection | |
| *Representativeness of the exposed cohort？* | *** |
| *Selection of the non exposed cohort* | *** |
| *Ascertainment of exposure* | *** |
| *Demonstration that outcome of interest was not present at start of study* | *** |
| Comparability | |
| *Comparability of cohorts on the basis of the design or analysis* | *** |
| Outcome | |
| *Assessment of outcome* | *** |
| *Was follow-up long enough for outcomes to occur* | *** |
| *Adequacy of follow up of cohorts* |  |

Studies with scores of 0-3, 4-6, 7-9 were, respectively, considered as low, moderate, and high quality.

High quality

| **Study name:** Giri, Abhishek R.****** | |
| --- | --- |
| **Items** | **Response options** |
| Selection | |
| *Representativeness of the exposed cohort？* | *** |
| *Selection of the non exposed cohort* |  |
| *Ascertainment of exposure* | *** |
| *Demonstration that outcome of interest was not present at start of study* | *** |
| Comparability | |
| *Comparability of cohorts on the basis of the design or analysis* |  |
| Outcome | |
| *Assessment of outcome* | *** |
| *Was follow-up long enough for outcomes to occur* |  |
| *Adequacy of follow up of cohorts* |  |

Studies with scores of 0-3, 4-6, 7-9 were, respectively, considered as low, moderate, and high quality.

Moderate quality

| **Study name:** Bagate, F. | |
| --- | --- |
| **Items** | **Response options** |
| Selection | |
| *Representativeness of the exposed cohort？* | *** |
| *Selection of the non exposed cohort* |  |
| *Ascertainment of exposure* | *** |
| *Demonstration that outcome of interest was not present at start of study* | *** |
| Comparability | |
| *Comparability of cohorts on the basis of the design or analysis* |  |
| Outcome | |
| *Assessment of outcome* | *** |
| *Was follow-up long enough for outcomes to occur* |  |
| *Adequacy of follow up of cohorts* |  |

Studies with scores of 0-3, 4-6, 7-9 were, respectively, considered as low, moderate, and high quality.

Moderate quality

| **Study name:** Caplan, M.****** | |
| --- | --- |
| **Items** | **Response options** |
| Selection | |
| *Representativeness of the exposed cohort？* | *** |
| *Selection of the non exposed cohort* |  |
| *Ascertainment of exposure* | *** |
| *Demonstration that outcome of interest was not present at start of study* | *** |
| Comparability | |
| *Comparability of cohorts on the basis of the design or analysis* |  |
| Outcome | |
| *Assessment of outcome* | *** |
| *Was follow-up long enough for outcomes to occur* | *** |
| *Adequacy of follow up of cohorts* | *** |

Studies with scores of 0-3, 4-6, 7-9 were, respectively, considered as low, moderate, and high quality.

Moderate quality

| **Study name:** Saccheri, C.****** | |
| --- | --- |
| **Items** | **Response options** |
| Selection | |
| *Representativeness of the exposed cohort？* | *** |
| *Selection of the non exposed cohort* |  |
| *Ascertainment of exposure* | *** |
| *Demonstration that outcome of interest was not present at start of study* | *** |
| Comparability | |
| *Comparability of cohorts on the basis of the design or analysis* |  |
| Outcome | |
| *Assessment of outcome* | *** |
| *Was follow-up long enough for outcomes to occur* | *** |
| *Adequacy of follow up of cohorts* | *** |

Studies with scores of 0-3, 4-6, 7-9 were, respectively, considered as low, moderate, and high quality.

Moderate quality

| **Study name:** Cardinale, M. | |
| --- | --- |
| **Items** | **Response options** |
| Selection | |
| *Representativeness of the exposed cohort？* | *** |
| *Selection of the non exposed cohort* |  |
| *Ascertainment of exposure* | *** |
| *Demonstration that outcome of interest was not present at start of study* |  |
| Comparability | |
| *Comparability of cohorts on the basis of the design or analysis* |  |
| Outcome | |
| *Assessment of outcome* | *** |
| *Was follow-up long enough for outcomes to occur* | *** |
| *Adequacy of follow up of cohorts* | *** |

Studies with scores of 0-3, 4-6, 7-9 were, respectively, considered as low, moderate, and high quality.

Moderate quality

| **Study name:** Al Sulaiman, K. | |
| --- | --- |
| **Items** | **Response options** |
| Selection | |
| *Representativeness of the exposed cohort？* | *** |
| *Selection of the non exposed cohort* | *** |
| *Ascertainment of exposure* | *** |
| *Demonstration that outcome of interest was not present at start of study* |  |
| Comparability | |
| *Comparability of cohorts on the basis of the design or analysis* | *** |
| Outcome | |
| *Assessment of outcome* | *** |
| *Was follow-up long enough for outcomes to occur* | *** |
| *Adequacy of follow up of cohorts* | *** |

Studies with scores of 0-3, 4-6, 7-9 were, respectively, considered as low, moderate, and high quality.

High quality

| **Study name:** Abou-Arab, O. | |
| --- | --- |
| **Items** | **Response options** |
| Selection | |
| *Representativeness of the exposed cohort？* | *** |
| *Selection of the non exposed cohort* | *** |
| *Ascertainment of exposure* | *** |
| *Demonstration that outcome of interest was not present at start of study* |  |
| Comparability | |
| *Comparability of cohorts on the basis of the design or analysis* |  |
| Outcome | |
| *Assessment of outcome* | *** |
| *Was follow-up long enough for outcomes to occur* | *** |
| *Adequacy of follow up of cohorts* | *** |

Studies with scores of 0-3, 4-6, 7-9 were, respectively, considered as low, moderate, and high quality.

Moderate quality

| **Study name:** Chandel, A. | |
| --- | --- |
| **Items** | **Response options** |
| Selection | |
| *Representativeness of the exposed cohort？* | *** |
| *Selection of the non exposed cohort* | *** |
| *Ascertainment of exposure* | *** |
| *Demonstration that outcome of interest was not present at start of study* |  |
| Comparability | |
| *Comparability of cohorts on the basis of the design or analysis* |  |
| Outcome | |
| *Assessment of outcome* | *** |
| *Was follow-up long enough for outcomes to occur* | *** |
| *Adequacy of follow up of cohorts* | *** |

Studies with scores of 0-3, 4-6, 7-9 were, respectively, considered as low, moderate, and high quality.

Moderate quality

| **Study name:** Safaee Fakhr, B. | |
| --- | --- |
| **Items** | **Response options** |
| Selection | |
| *Representativeness of the exposed cohort？* | *** |
| *Selection of the non exposed cohort* |  |
| *Ascertainment of exposure* | *** |
| *Demonstration that outcome of interest was not present at start of study* |  |
| Comparability | |
| *Comparability of cohorts on the basis of the design or analysis* |  |
| Outcome | |
| *Assessment of outcome* | *** |
| *Was follow-up long enough for outcomes to occur* | *** |
| *Adequacy of follow up of cohorts* | *** |

Studies with scores of 0-3, 4-6, 7-9 were, respectively, considered as low, moderate, and high quality.

Moderate quality

| **Study name:** Barthélémy, R. | |
| --- | --- |
| **Items** | **Response options** |
| Selection | |
| *Representativeness of the exposed cohort？* | *** |
| *Selection of the non exposed cohort* |  |
| *Ascertainment of exposure* | *** |
| *Demonstration that outcome of interest was not present at start of study* |  |
| Comparability | |
| *Comparability of cohorts on the basis of the design or analysis* |  |
| Outcome | |
| *Assessment of outcome* | *** |
| *Was follow-up long enough for outcomes to occur* |  |
| *Adequacy of follow up of cohorts* | *** |

Studies with scores of 0-3, 4-6, 7-9 were, respectively, considered as low, moderate, and high quality.

Moderate quality

| **Study name:** Ziehr, D. R. | |
| --- | --- |
| **Items** | **Response options** |
| Selection | |
| *Representativeness of the exposed cohort？* | *** |
| *Selection of the non exposed cohort* | *** |
| *Ascertainment of exposure* | *** |
| *Demonstration that outcome of interest was not present at start of study* |  |
| Comparability | |
| *Comparability of cohorts on the basis of the design or analysis* |  |
| Outcome | |
| *Assessment of outcome* | *** |
| *Was follow-up long enough for outcomes to occur* |  |
| *Adequacy of follow up of cohorts* | *** |

Studies with scores of 0-3, 4-6, 7-9 were, respectively, considered as low, moderate, and high quality.

Moderate quality

| **Study name:** Lotz, C. | |
| --- | --- |
| **Items** | **Response options** |
| Selection | |
| *Representativeness of the exposed cohort？* | *** |
| *Selection of the non exposed cohort* | *** |
| *Ascertainment of exposure* | *** |
| *Demonstration that outcome of interest was not present at start of study* |  |
| Comparability | |
| *Comparability of cohorts on the basis of the design or analysis* |  |
| Outcome | |
| *Assessment of outcome* | *** |
| *Was follow-up long enough for outcomes to occur* | *** |
| *Adequacy of follow up of cohorts* | *** |

Studies with scores of 0-3, 4-6, 7-9 were, respectively, considered as low, moderate, and high quality.

Moderate quality

| **Study name:** Mekontso Dessap, A. | |
| --- | --- |
| **Items** | **Response options** |
| Selection | |
| *Representativeness of the exposed cohort？* | *** |
| *Selection of the non exposed cohort* | *** |
| *Ascertainment of exposure* | *** |
| *Demonstration that outcome of interest was not present at start of study* |  |
| Comparability | |
| *Comparability of cohorts on the basis of the design or analysis* |  |
| Outcome | |
| *Assessment of outcome* | *** |
| *Was follow-up long enough for outcomes to occur* | *** |
| *Adequacy of follow up of cohorts* | *** |

Studies with scores of 0-3, 4-6, 7-9 were, respectively, considered as low, moderate, and high quality.

Moderate quality

| **Study name:** Di Fenza, R. | |
| --- | --- |
| **Items** | **Response options** |
| Selection | |
| *Representativeness of the exposed cohort？* | *** |
| *Selection of the non exposed cohort* | *** |
| *Ascertainment of exposure* | *** |
| *Demonstration that outcome of interest was not present at start of study* |  |
| Comparability | |
| *Comparability of cohorts on the basis of the design or analysis* | *** |
| Outcome | |
| *Assessment of outcome* | *** |
| *Was follow-up long enough for outcomes to occur* | *** |
| *Adequacy of follow up of cohorts* | *** |

Studies with scores of 0-3, 4-6, 7-9 were, respectively, considered as low, moderate, and high quality.

High quality

| **Study name:** Blot, P. L. | |
| --- | --- |
| **Items** | **Response options** |
| Selection | |
| *Representativeness of the exposed cohort？* | *** |
| *Selection of the non exposed cohort* | *** |
| *Ascertainment of exposure* | *** |
| *Demonstration that outcome of interest was not present at start of study* |  |
| Comparability | |
| *Comparability of cohorts on the basis of the design or analysis* |  |
| Outcome | |
| *Assessment of outcome* | *** |
| *Was follow-up long enough for outcomes to occur* | *** |
| *Adequacy of follow up of cohorts* | *** |

Studies with scores of 0-3, 4-6, 7-9 were, respectively, considered as low, moderate, and high quality.

Moderate quality

**Supplementary 2 table** 2**. NOS assessment results of case-control studies**

| **Study name:** **Longobardo, A.** | |
| --- | --- |
| **Items** | **Response options** |
| Selection | |
| *Is the case definition adequate?？* | *** |
| *Representativeness of the cases* | *** |
| *Selection of controls* |  |
| *Definition of controls* | *** |
| Comparability | |
| *Comparability of cohorts on the basis of the design or analysis* |  |
| Exposure | |
| *Ascertainment of exposure* | *** |
| *Same method of ascertainment for cases and controls* | *** |
| *Non-Response rate* |  |

Studies with scores of 0-3, 4-6, 7-9 were, respectively, considered as low, moderate, and high quality.

Moderate quality

We arbitrarily defined the study at high quality if it explicitly described identification of the condition for all participants, clearly reported clinical information of the participants and the outcomes or follow up results of cases, and all other items were assessed as Yes or NA; at low quality if it were not met all three criteria, regardless of assessment of other items; at moderate quality if it did not meet criteria for high or low quality.

### Supplementary 2 table 3. JBI assessment results of case reports

| **Study name: Heuts, S.** | |
| --- | --- |
| **Items** | **Response options** |
| *1.Were patient’s demographic characteristics clearly described?* | **Y** |
| *2.Was the patient’s history clearly described and presented as a timeline?* | **N** |
| *3.Was the current clinical condition of the patient on presentation clearly described?* | **Y** |
| *4.Were diagnostic tests or assessment methods and the results clearly described?* | **Y** |
| *5.Was the intervention(s) or treatment procedure(s) clearly described?* | **N** |
| *6.Was the post-intervention clinical condition clearly described?* | **Y** |
| *7.Were adverse events (harms) or unanticipated events identified and described?* | **NA** |
| *8.Does the case report provide takeaway lessons?* | **Y** |

Moderate quality

| **Study name: Brown, C. J.** | |
| --- | --- |
| **Items** | **Response options** |
| *1.Were patient’s demographic characteristics clearly described?* | **Y** |
| *2.Was the patient’s history clearly described and presented as a timeline?* | **N** |
| *3.Was the current clinical condition of the patient on presentation clearly described?* | **Y** |
| *4.Were diagnostic tests or assessment methods and the results clearly described?* | **Y** |
| *5.Was the intervention(s) or treatment procedure(s) clearly described?* | **N** |
| *6.Was the post-intervention clinical condition clearly described?* | **Y** |
| *7.Were adverse events (harms) or unanticipated events identified and described?* | **NA** |
| *8.Does the case report provide takeaway lessons?* | **Y** |

Moderate quality

| **Study name:** Huette, P.^a^ | |
| --- | --- |
| **Items** | **Response options** |
| *1.Were patient’s demographic characteristics clearly described?* | **Y** |
| *2.Was the patient’s history clearly described and presented as a timeline?* | **Y** |
| *3.Was the current clinical condition of the patient on presentation clearly described?* | **Y** |
| *4.Were diagnostic tests or assessment methods and the results clearly described?* | **Y** |
| *5.Was the intervention(s) or treatment procedure(s) clearly described?* | **Y** |
| *6.Was the post-intervention clinical condition clearly described?* | **Y** |
| *7.Were adverse events (harms) or unanticipated events identified and described?* | **NA** |
| *8.Does the case report provide takeaway lessons?* | **Y** |

High quality

| **Study name:** Huette, P.^b^ | |
| --- | --- |
| **Items** | **Response options** |
| *1.Were patient’s demographic characteristics clearly described?* | **Y** |
| *2.Was the patient’s history clearly described and presented as a timeline?* | **Y** |
| *3.Was the current clinical condition of the patient on presentation clearly described?* | **Y** |
| *4.Were diagnostic tests or assessment methods and the results clearly described?* | **Y** |
| *5.Was the intervention(s) or treatment procedure(s) clearly described?* | **Y** |
| *6.Was the post-intervention clinical condition clearly described?* | **Y** |
| *7.Were adverse events (harms) or unanticipated events identified and described?* | **Y** |
| *8.Does the case report provide takeaway lessons?* | **Y** |

High quality

| **Study name:** Paramanathan, S. | |
| --- | --- |
| **Items** | **Response options** |
| *1.Were patient’s demographic characteristics clearly described?* | **Y** |
| *2.Was the patient’s history clearly described and presented as a timeline?* | **Y** |
| *3.Was the current clinical condition of the patient on presentation clearly described?* | **Y** |
| *4.Were diagnostic tests or assessment methods and the results clearly described?* | **Y** |
| *5.Was the intervention(s) or treatment procedure(s) clearly described?* | **Y** |
| *6.Was the post-intervention clinical condition clearly described?* | **Y** |
| *7.Were adverse events (harms) or unanticipated events identified and described?* | **NA** |
| *8.Does the case report provide takeaway lessons?* | **Y** |

High quality

| **Study name:** Vives, M. | |
| --- | --- |
| **Items** | **Response options** |
| *1.Were patient’s demographic characteristics clearly described?* | **Y** |
| *2.Was the patient’s history clearly described and presented as a timeline?* | **N** |
| *3.Was the current clinical condition of the patient on presentation clearly described?* | **Y** |
| *4.Were diagnostic tests or assessment methods and the results clearly described?* | **Y** |
| *5.Was the intervention(s) or treatment procedure(s) clearly described?* | **Y** |
| *6.Was the post-intervention clinical condition clearly described?* | **Y** |
| *7.Were adverse events (harms) or unanticipated events identified and described?* | **NA** |
| *8.Does the case report provide takeaway lessons?* | **Y** |

Moderate quality

| **Study name:** Feng, W. X. | |
| --- | --- |
| **Items** | **Response options** |
| *1.Were patient’s demographic characteristics clearly described?* | **Y** |
| *2.Was the patient’s history clearly described and presented as a timeline?* | **Y** |
| *3.Was the current clinical condition of the patient on presentation clearly described?* | **Y** |
| *4.Were diagnostic tests or assessment methods and the results clearly described?* | **Y** |
| *5.Was the intervention(s) or treatment procedure(s) clearly described?* | **Y** |
| *6.Was the post-intervention clinical condition clearly described?* | **Y** |
| *7.Were adverse events (harms) or unanticipated events identified and described?* | **NA** |
| *8.Does the case report provide takeaway lessons?* | **Y** |

High quality

| **Study name:** van Zyl, A. G. P. | |
| --- | --- |
| **Items** | **Response options** |
| *1.Were patient’s demographic characteristics clearly described?* | **Y** |
| *2.Was the patient’s history clearly described and presented as a timeline?* | **Y** |
| *3.Was the current clinical condition of the patient on presentation clearly described?* | **Y** |
| *4.Were diagnostic tests or assessment methods and the results clearly described?* | **Y** |
| *5.Was the intervention(s) or treatment procedure(s) clearly described?* | **Y** |
| *6.Was the post-intervention clinical condition clearly described?* | **Y** |
| *7.Were adverse events (harms) or unanticipated events identified and described?* | **NA** |
| *8.Does the case report provide takeaway lessons?* | **Y** |

High quality

We arbitrarily defined the study at high quality if it explicitly described identification of the condition for all participants, clearly reported clinical information of the participants and the outcomes or follow up results of cases, and all other items were assessed as Yes or NA; at low quality if it were not met all three criteria, regardless of assessment of other items; at moderate quality if it did not meet criteria for high or low quality.

### Supplementary 2 table 4. JBI assessment results of case series

| **Study name: Losser, MR** | |
| --- | --- |
| **Items** | **Response options** |
| *1.* *Were there clear criteria for inclusion in the case series?* | **Y** |
| *2.* *Was the condition measured in a standard, reliable way for all participants included in the case series?* | **Y** |
| *3.* *Were valid methods used for identification of the condition for all participants included in the case series?* | **Y** |
| *4.* *Did the case series have consecutive inclusion of participants?* | **Y** |
| *5.* *Did the case series have complete inclusion of participants?* | **Y** |
| *6.* *Was there clear reporting of the demographics of the participants in the study?* | **Y** |
| *7.* *Was there clear reporting of clinical information of the participants?* | **Y** |
| *8.* *Were the outcomes or follow up results of cases clearly reported?* | **Y** |
| *9.Was there clear reporting of the presenting site(s)-clinic(s) demographic information?* | **N** |
| *10.* *Was statistical analysis appropriate?* | **Y** |

Moderate quality

| **Study name: Ferrari, M.** | |
| --- | --- |
| **Items** | **Response options** |
| *1.* *Were there clear criteria for inclusion in the case series?* | **Y** |
| *2.* *Was the condition measured in a standard, reliable way for all participants included in the case series?* | **N** |
| *3.* *Were valid methods used for identification of the condition for all participants included in the case series?* | **Y** |
| *4.* *Did the case series have consecutive inclusion of participants?* | **Y** |
| *5.* *Did the case series have complete inclusion of participants?* | **Y** |
| *6.* *Was there clear reporting of the demographics of the participants in the study?* | **Y** |
| *7.* *Was there clear reporting of clinical information of the participants?* | **Y** |
| *8.* *Were the outcomes or follow up results of cases clearly reported?* | **Y** |
| *9.Was there clear reporting of the presenting site(s)-clinic(s) demographic information?* | **N** |
| *10.* *Was statistical analysis appropriate?* | **Y** |

Moderate quality

| **Study name: Tavazzi, G.** | |
| --- | --- |
| **Items** | **Response options** |
| *1.* *Were there clear criteria for inclusion in the case series?* | **Y** |
| *2.* *Was the condition measured in a standard, reliable way for all participants included in the case series?* | **N** |
| *3.* *Were valid methods used for identification of the condition for all participants included in the case series?* | **Y** |
| *4.* *Did the case series have consecutive inclusion of participants?* | **Y** |
| *5.* *Did the case series have complete inclusion of participants?* | **Y** |
| *6.* *Was there clear reporting of the demographics of the participants in the study?* | **Y** |
| *7.* *Was there clear reporting of clinical information of the participants?* | **Y** |
| *8.* *Were the outcomes or follow up results of cases clearly reported?* | **Y** |
| *9.Was there clear reporting of the presenting site(s)-clinic(s) demographic information?* | **N** |
| *10.* *Was statistical analysis appropriate?* | **Y** |

Moderate quality

| **Study name: Bicakcioglu, M.** | |
| --- | --- |
| **Items** | **Response options** |
| *1.* *Were there clear criteria for inclusion in the case series?* | **Y** |
| *2.* *Was the condition measured in a standard, reliable way for all participants included in the case series?* | **N** |
| *3.* *Were valid methods used for identification of the condition for all participants included in the case series?* | **Y** |
| *4.* *Did the case series have consecutive inclusion of participants?* | **Y** |
| *5.* *Did the case series have complete inclusion of participants?* | **Y** |
| *6.* *Was there clear reporting of the demographics of the participants in the study?* | **Y** |
| *7.* *Was there clear reporting of clinical information of the participants?* | **Y** |
| *8.* *Were the outcomes or follow up results of cases clearly reported?* | **Y** |
| *9.Was there clear reporting of the presenting site(s)-clinic(s) demographic information?* | **N** |
| *10.* *Was statistical analysis appropriate?* | **Y** |

We arbitrarily defined the study at high quality if it clearly described study subjects and the setting, identified and deal with the confounding factors, and all other items were assessed as Yes or NA; at low quality if it were not met all three criteria, regardless of assessment of other items; at moderate quality if it did not meet criteria for high or low quality.

### Supplementary 2 table 5. JBI assessment results of cross-sectional study

| **Study name: Herranz, L.** | |
| --- | --- |
| **Items** | **Response options** |
| *1.* *Were the criteria for inclusion in the sample clearly defined?* | **Y** |
| *2.* *Were the study subjects and the setting described in detail?* | **Y** |
| *3.* *Was the exposure measured in a valid and reliable way?* | **Y** |
| *4.* *Were objective, standard criteria used for measurement of the condition?* | **Y** |
| *5.* *Were confounding factors identified?* | **N** |
| *6.* *Were strategies to deal with confounding factors stated?* | **N** |
| *7.* *Were the outcomes measured in a valid and reliable way?* | **Y** |
| *8.* *Was appropriate statistical analysis used?* | **Y** |

Moderate quality

**Supplementary 2 figure S3-6.**


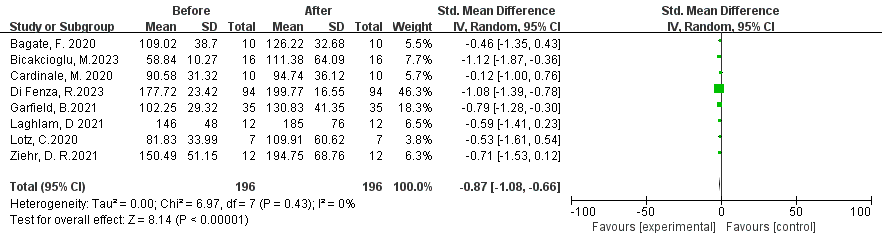


Figure S3 P/F before and after administration of inhalation NO


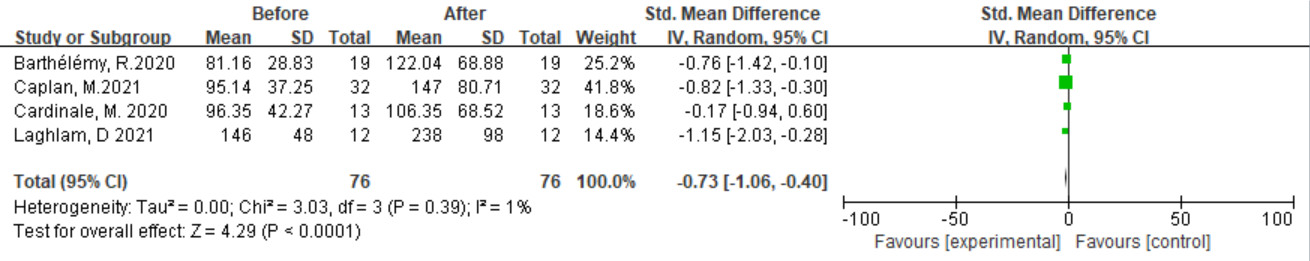


Figure S4 P/F before and after administration of almitrine


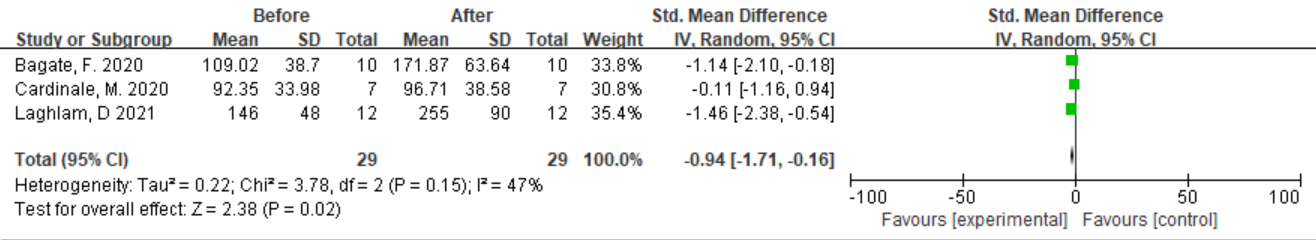


Figure S5 P/F before and after administration of NO combined with almitrine


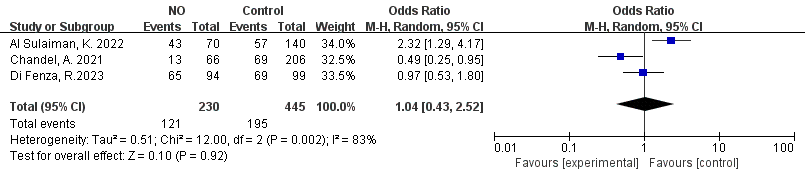


Figure S6 The SAEs of inhalation NO for COVID-19(at the edge of sepsis)

**Supplementary 2 Table 6: Grading of Recommendations, Assessments, Developments, and Evaluations (GRADE) Approach for assessing certainty of evidence**

| **Quality assessment** | | | | | | | **No of patients** | | **Effect** | | **Quality** | **Importance** |  |
| --- | --- | --- | --- | --- | --- | --- | --- | --- | --- | --- | --- | --- | --- |
|  |  |  |  |  |  |  |  |  |  |  |  |  |  |
| **No of studies** | **Design** | **Risk of bias** | **Inconsistency** | **Indirectness** | **Imprecision** | **Other considerations** | **COVID-19(at the edge of sepsis)- dichotomous** | **Control** | **Relative (95% CI)** | **Absolute** |  |  |  |
| **NO for COVID-19(at the edge of sepsis)-28day mortality** | | | | | | | | | | | | |  |
| 4 | observational studies | no serious risk of bias | serious^1^ | no serious indirectness | serious^2^ | none | 89/194  (45.9%) | 70/228  (30.7%) | OR 0.72 (0.14 to 3.7) | 65 fewer per 1000 (from 249 fewer to 314 more) | ⊕OOO VERY LOW | CRITICAL |  |
|  |  |  |  |  |  |  |  | 33.9% |  | 69 fewer per 1000 (from 272 fewer to 316 more) |  |  |  |
| **almitrine for COVID-19(at the edge of sepsis)-28day mortality** | | | | | | | | | | | | |  |
| 1 | randomised trials | no serious risk of bias | serious^3^ | no serious indirectness | serious^4^ | none^4^ | 7/88  (8%) | 15/91  (16.5%) | OR 0.44 (0.17 to 1.13) | 85 fewer per 1000 (from 132 fewer to 18 more) | ⊕⊕OO LOW | CRITICAL |  |
|  |  |  |  |  |  |  |  | 16.5% |  | 85 fewer per 1000 (from 132 fewer to 18 more) |  |  |  |
| **NO for COVID-19(at the edge of sepsis)-in hospital mortality** | | | | | | | | | | | | |  |
| 3 | observational studies | no serious risk of bias | serious^1^ | no serious indirectness | serious^2^ | none | 88/177  (49.7%) | 144/408  (35.3%) | OR 1.14 (0.39 to 3.32) | 30 more per 1000 (from 178 fewer to 291 more) | ⊕OOO VERY LOW | CRITICAL |  |
|  |  |  |  |  |  |  |  | 37.1% |  | 31 more per 1000 (from 184 fewer to 291 more) |  |  |  |
| **almitrine for COVID-19(at the edge of sepsis)-in hospital mortality** | | | | | | | | | | | | |  |
| 1 | randomised trials | no serious risk of bias | serious^3^ | no serious indirectness | serious^4^ | none | 7/88  (8%) | 15/91  (16.5%) | OR 0.44 (0.17 to 1.13) | 85 fewer per 1000 (from 132 fewer to 18 more) | ⊕⊕OO LOW | CRITICAL |  |
|  |  |  |  |  |  |  |  | 16.5% |  | 85 fewer per 1000 (from 132 fewer to 18 more) |  |  |  |
| **NO for COVID-19(at the edge of sepsis)-needs for intubation** | | | | | | | | | | | | |  |
| 3 | observational studies | no serious risk of bias | serious^5^ | no serious indirectness | serious^2^ | none | 52/121  (43%) | 122/279  (43.7%) | OR 0.82 (0.34 to 1.93) | 48 fewer per 1000 (from 228 fewer to 163 more) | ⊕OOO VERY LOW | IMPORTANT |  |
|  |  |  |  |  |  |  |  | 38.4% |  | 46 fewer per 1000 (from 209 fewer to 162 more) |  |  |  |
| **almitrine for COVID-19(at the edge of sepsis)-needs for intubation** | | | | | | | | | | | | |  |
| 1 | randomised trials | no serious risk of bias | serious^3^ | no serious indirectness | serious^2^ | none | 26/88  (29.5%) | 28/91  (30.8%) | OR 0.94 (0.5 to 1.79) | 13 fewer per 1000 (from 126 fewer to 135 more) | ⊕⊕OO LOW | IMPORTANT |  |
|  |  |  |  |  |  |  |  | 30.8% |  | 13 fewer per 1000 (from 126 fewer to 135 more) |  |  |  |
|  |  |  |  |  |  |  |  | 37.1% |  | 16 more per 1000 (from 252 fewer to 374 more) |  |  |  |
| **almitrine for COVID-19(at the edge of sepsis)-SAE** | | | | | | | | | | | | |  |
| 1 | randomised trials | no serious risk of bias | serious^3^ | no serious indirectness | serious^4^ | none | 32/88  (36.4%) | 30/91  (33%) | OR 1.16 (0.63 to 2.15) | 34 more per 1000 (from 93 fewer to 184 more) | ⊕⊕OO LOW | IMPORTANT |  |
|  |  |  |  |  |  |  |  | 33% |  | 34 more per 1000 (from 93 fewer to 184 more) |  |  |  |
| **NO+almitrine for COVID-19(at the edge of sepsis)-P/F before and after** | | | | | | | | | | | | |  |
| 3 | observational studies | no serious risk of bias | no serious inconsistency | no serious indirectness | Serious^2^ | none | 29 | 29 | - | SMD 0.94 lower (1.71 to 0.16 lower) | ⊕OOO VERY LOW | IMPORTANT |  |
| **NO for COVID-19(at the edge of sepsis)-P/F before and after** | | | | | | | | | | | | |  |
| 6 | observational studies | no serious risk of bias | no serious inconsistency | no serious indirectness | serious^2^ | none | 86 | 86 | - | SMD 0.61 lower (0.91 to 0.3 lower) | ⊕OOO VERY LOW | IMPORTANT |  |
| **almitrine for COVID-19(at the edge of sepsis)-P/F before and after** | | | | | | | | | | | | |  |
| 4 | observational studies | no serious risk of bias | no serious inconsistency | no serious indirectness | serious^2^ | none | 76 | 76 | - | SMD 0.73 lower (1.06 to 0.4 lower) | ⊕OOO VERY LOW | IMPORTANT |  |
| **NO for COVID-19(at the edge of sepsis) Hospital length of stay** | | | | | | | | | | | | |  |
| 3 | observational studies | no serious risk of bias | serious^1^ | no serious indirectness | serious^2^ | none | 150 | 357 | - | MD 8.58 higher (3.07 lower to 20.23 higher) | ⊕OOO VERY LOW | IMPORTANT |  |
| **almitrine for COVID-19(at the edge of sepsis) Hospital length of stay** | | | | | | | | | | | | |  |
| 1 | randomised trials | no serious risk of bias | serious^3^ | no serious indirectness | serious^4^ | none | 88 | 91 | - | SMD 0 higher (0.29 lower to 0.29 higher) | ⊕⊕OO LOW | IMPORTANT |  |

^1^ There was important heterogeneity.

^2^ There were too few studies to evaluate the information size and results, so we downgraded one point for imprecision.
^3^ With only one RCT, heterogeneity could not be calculated.
^4^ There were too few randomized controlled studies to evaluate the information size and results, so we downgraded one point for imprecision.
^5^ There was some heterogeneity (I^2^ = 50-60%) in the point estimates.
^6^ There were too few studies to evaluate the information size and results and the 95% CI is relatively wide.
