# Supplementary material for: Unraveling the impact of nitric oxide, almitrine, and their combination in COVID-19 (at the edge of sepsis) patients: a systematic review
Source: Front Pharmacol. 2024 Jan 22;14:1172447. doi: 10.3389/fphar.2023.1172447 (PMC10839063; doi:10.3389/fphar.2023.1172447)
Supplement: Supplementary file 2 [file DataSheet1.docx]

**Supplementary 1**

| Supplementary 1 appendix | Page |
| --- | --- |
| Supplementary 1 table 1. PRISMA Checklist | **1-8** |
| Supplementary 1 table 2-4. Search strategy | **9-11** |
| Supplementary 1 table 5. List of excluded studies | **12-13** |
| Supplementary 1. References of excluded studies | **14-16** |
| Supplementary 1. References of included studies | **17-18** |

**Supplementary 1 table 1. PRISMA Checklist**

| **Section and Topic** | **Item #** | **Checklist item** | **Location where item is reported** |
| --- | --- | --- | --- |
| **TITLE** | | |  |
| Title | 1 | Unravelling the Impact of Nitric Oxide, Almitrine, and their Combination in COVID-19 (at the edge of sepsis) patients: A systematic review | Title |
| **ABSTRACT** | | |  |
| Abstract | 2 | During the coronavirus disease 2019 (COVID-19) pandemic, a large number of severe and critically ill COVID-19 patients meet the diagnostic criteria for sepsis and septic shock. The treatment for COVID-19 patients with sepsis is still very limited. For sepsis, improving ventilation is the main treatment. Nitric oxide(NO) and almitrine have been reported to improve oxygenation in patients with “classical” sepsis. Here, we conducted a systematic review and meta-analysis to evaluate the efficacy and safety of NO, almitrine and combination of both for COVID-19(at the edge of sepsis) patients. | Abstract |
| **INTRODUCTION** | | |  |
| Rationale | 3 | Coronavirus disease 2019 (COVID-19) affects every aspect of human life by challenging bodily, socioeconomic, and political systems at unprecedented levels. As vaccines become available, their distribution, safety, and efficacy against emerging variants remain uncertain, and specific treatments are lacking. As of 2 October 2022, more than 615 million people have been diagnosed with covid-19 and more than 6.5 million deaths have occurred globally. Although the disease is asymptomatic or mild in most patients, a substantial percentage of people have more extensive pneumonia that can progress to hypoxaemic respiratory failure, shock, dysfunction of organs, and death. Sepsis is a syndrome of physiologic, pathologic and bio-chemical abnormalities induced by infection. As a result of the COVID-19 pandemic, the reported incidence of sepsis is increasing. However, mechanisms underlying COVID-19(at the edge of sepsis) are incompletely understood.  Inhaled nitric oxide (INO) is a gaseous free radical which is produced from arginine with the help of enzymes (neuronal, endothelial, and inducible nitric oxide synthase) that controls vasodilation. The INO plays a specific role in maintaining the vascular system and has the unique capability to produce pulmonary vasodilation by involving in the pathological and physiological process, which includes relaxation of smooth muscle cells, immune response, and antimicrobial activities to increase the blood flow. In addition to its favorable pulmonary vascular effects, in-vitro studies report that nitric oxide donors can inhibit replication of viruses, including SARS Coronavirus 2 (SARS-CoV-2). We aim to systematically appraise the literature to assess the safety and effectiveness of INO for the treatment of COVID-19(at the edge of sepsis). | Introduction |
| Objectives | 4 | This study is the first to screen COVID-19 patients with sepsis or at the edge of sepsis through SOFA score, and the efficacy and safety of nitric oxide, almitrine, and the combination of both in COVID-19 patients with organ dysfunction (SOFA≥2) were systematically reviewed.  Intervention(s)：nitric oxide, almitrine, and the combination of both  Comparator(s)/control: Placebo, standard treatment,  Outcome(s): mortality, intubation needs, ICU stay,and serious adverse events.  Study: Randomized clinical trials, cohort studies, case-control studies, cross sectional, case series, case reports, any other grey literature will be included. | Introduction |
| **METHODS** | | |  |
| Eligibility criteria | 5 | Inclusion criteria: (1) Patients were confirmed COVID-19 and their SOFA score (include median, mean value, and absolute value)≥2, or according to the SOFA scoring tool, a certain system index (include median, mean, and absolute value) should be within the range corresponding to the system score≥2, for example, PaO2/FiO2 ratio (P/F) (include absolute value, median value, or mean value) was less than 300 mmHg. SpO2/FiO2 ratio (S/F) of 315 corresponded with a P/F ratio of 300mmHg [S/F=64+0.84*(P/F)]. In this review, we defined such COVID-19 patients were at the edge of sepsis. (2) The intervention of interest was inhaled NO, intravenous almitrine, inhaled NO combined with intravenous almitrine with or without standard treatment. Comparator treatments included placebo, standard treatment, or no intervention; studies with no comparator group were also included. (3) Randomized clinical trials (RCTs), cohort studies, case-control studies, cross sectional, case series, case reports study and any other grey literatures will be included. The language will be limited to Chinese and English. Exclusion criteria were: (1) Patients were not diagnosed as SARS-CoV-2 infection; (2) The patients’ SOFA score (absolute value, median value, or mean value) were less than 2 or did not reach 2 on any of the system indicators; (3) Data on SOFA score or certain indicators in the SOFA scoring tool were not available in the text, supplementary materials, or any other relevant resources. (4) Studies without an available full text or data were incomplete or unavailable, conference abstracts, posters, opinion articles, commentaries, and in vitro studies. The planned primary outcome was 28-30 days mortality, in hospital mortality, P/F, intubation needs. The safety outcomes was serious adverse events(SAEs) such as acute kidney injury. | Methods |
| Information sources | 6 | Electronic searches were carried out in PubMed, EMBASE, Cochrane Library, Web of Science, Wanfang Database and China National Knowledge Infrastructure (CNKI) until 23.10.2023. | Methods |
| Search strategy | 7 | The search strategy is attached (Appendix 9-11). | Methods |
| Selection process | 8 | Following the removal of duplicate entries, a three-stage screening process was followed to identify eligible records through the sequential examination of each title, abstract, and full text. Two reviewers screened each record, with provision for arbitration from a third reviewer. | Methods |
| Data collection process | 9 | Eligible articles were identified for inclusion by screening the titles, abstracts, and full text. Other relevant studies were manually screened by investigators from the reference list of included studies for further analysis. Two independent reviewers (Y.W and K.Z) carried out search in a standardized process, followed to identify eligible records through the sequential examination of each title, abstract, and full text. Disagreements were resolved by consensus and unresolved conflicts were decided by a third reviewer(D.L). | Methods |
| Data items | 10a | Data was extracted from the eligible studies using a template by two independent reviewers (Y.W and K.Z) and validated by a third (D.L). The following information was extracted: published year, authors, country of the study, study type, sample size, participant demographics, SOFA score, patients’ position, drug, dosage, route of administration, control group, mortality outcome, safety outcome, conclusion of authors. | Methods |
|  | 10b | Patients: COVID-19 patients with organ dysfunction (SOFA≥2)  Intervention(s)：nitric oxide, almitrine, and the combination of both  Comparator(s)/control: Placebo, standard treatment,  Outcome(s): mortality, intubation needs, ICU stay,and serious adverse events.  Study: Randomized clinical trials, cohort studies, case-control studies, cross sectional, case series, case reports, any other grey literature will be included. | Methods |
| Study risk of bias assessment | 11 | Included studies were assessed the potential bias by two reviewers (RL.L and LP.L)independently. The third researcher (CJ.W) was consulted for resolving any difference of opinion. The ‘Risk of Bias’ 2.0 tool was used to assess the RCTs. The methodological quality for case-control and cohort studies were assessed based on the Newcastle-Ottawa Scale(NOS). The methodological quality of the included case reports, case series and cross-sectional staudies were assessed based on JBI critical appraisal tools. | Methods |
| Effect measures | 12 | For dichotomous outcomes, the number of events and total number of participants in two groups were recorded. For continuous outcomes, the total number of participants, mean and standard deviation were recorded. | Methods |
| Synthesis methods | 13a | 28-30 days mortality, in hospital mortality, P/F, intubation needs, and SAEs. | Methods |
|  | 13b | If the authors reported median and interquartile range, we estimated the mean and standard deviation. We report odd’s ratio (OR) for dichotomous outcomes and standard mean differences (Std MD) for continuous outcomes. | Methods |
|  | 13c | One reviewer (Y.W) entered the data into the software, and another reviewer (RL.L) checked the data for accuracy. | Methods |
|  | 13d | The Review Manager version 5.4.1 software was used for analyses. Fixed-effects model was used if the result of the Q test was not significant (P>0.1) and I^2^<50%. | Methods |
|  | 13e | Subgroup analyses would be performed, if appropriate based on the data retrieved. | Methods |
|  | 13f | Due to lack of literature, sensitivity analysis cannot be carried out. | Methods |
| Reporting bias assessment | 14 | Due to lack of literature, sensitivity analysis cannot be carried out. | Methods |
| Certainty assessment | 15 | The quality of evidence was assessed by using the ‘Grading of Recommendations Assessment Development and Evaluation (GRADE)’ tool. | Methods |
| **RESULTS** | | |  |
| Study selection | 16a | Search of the electronic databases on 18rd Oct 2023 yielded a total of 83,090 studies. Following removing duplicates and screening of titles and abstracts, we evaluated 87 articles in full text. | Results |
|  | 16b | The list of excluded studies is attached (Appendix 14-16). | Results |
| Study characteristics | 17 | The references of included studies is attached (Appendix 17-18).. | Results |
| Risk of bias in studies | 18 | Displayed in Supplementary 2, appendix p1-30 | Results |
| Results of individual studies | 19 | 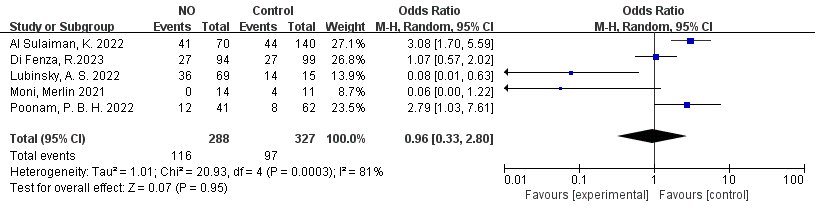  Figure S3 Mortality at 28-30 days of inhalation NO for COVID-19(at the edge of sepsis)  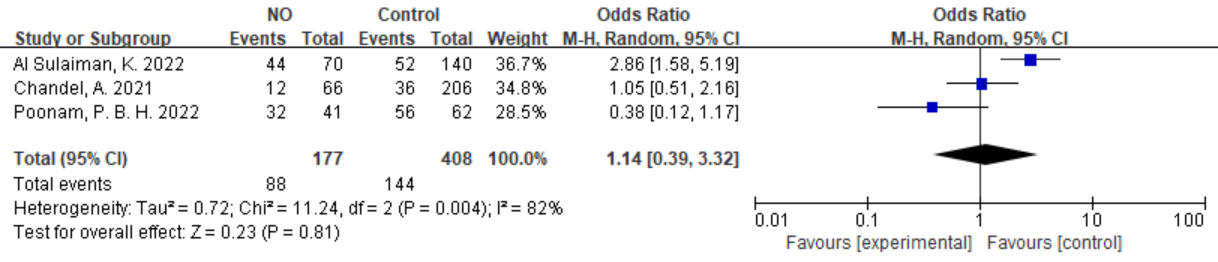  Figure S4 In hospital mortality of inhalation NO for COVID-19(at the edge of sepsis)  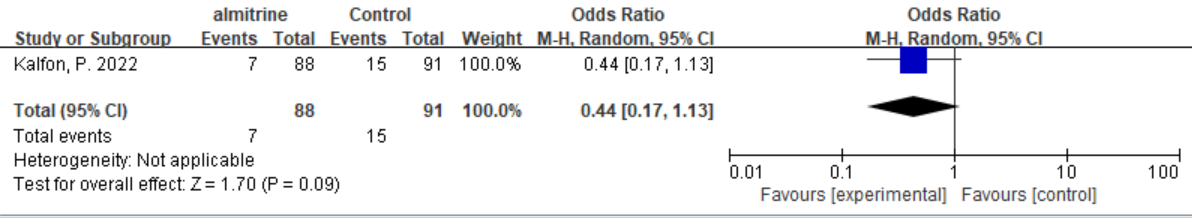  Figure S5 Mortality at 28-30 days of almitrine for COVID-19(at the edge of sepsis)  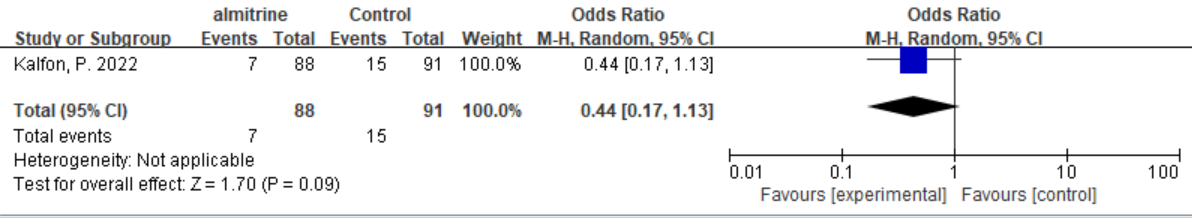  Figure S6 In hospital mortality of almitrine for COVID-19(at the edge of sepsis)  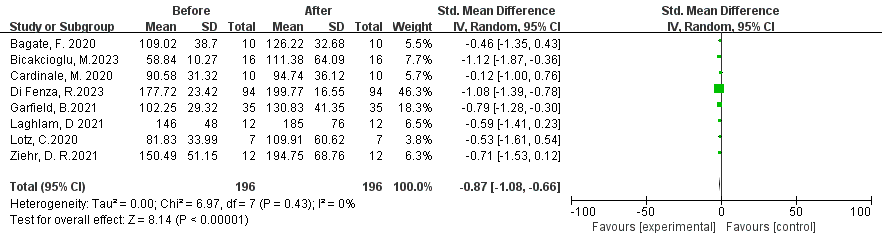  Figure S7 P/F before and after administration of inhalation NO  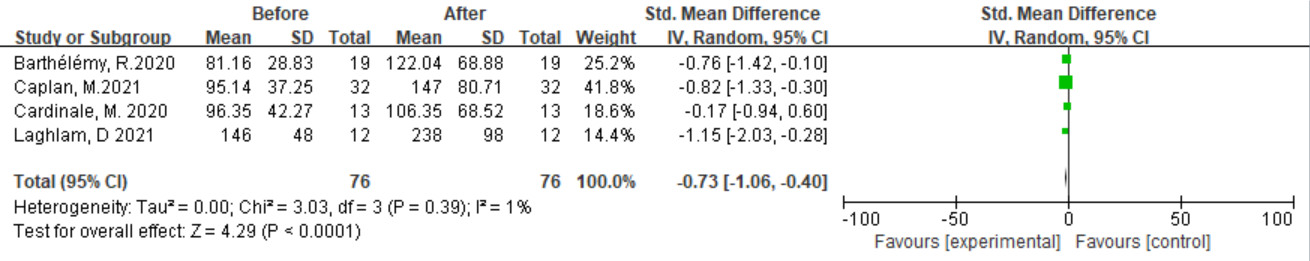  Figure S8 P/F before and after administration of almitrine  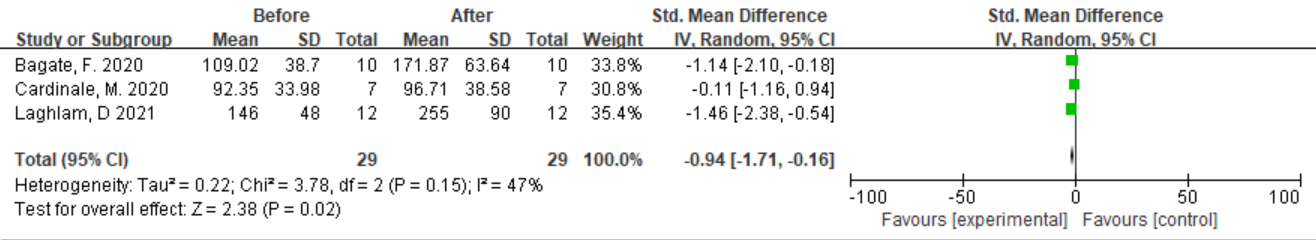  Figure S9 P/F before and after administration of NO combined with almitrine  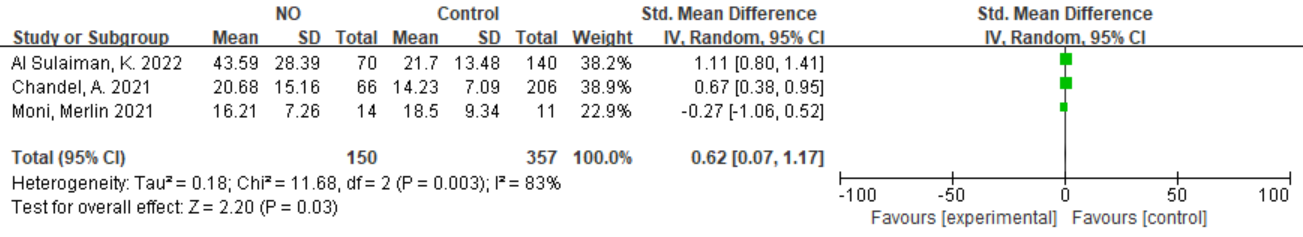  Figure S10 Hospital length of stay of inhalation NO for COVID-19(at the edge of sepsis)  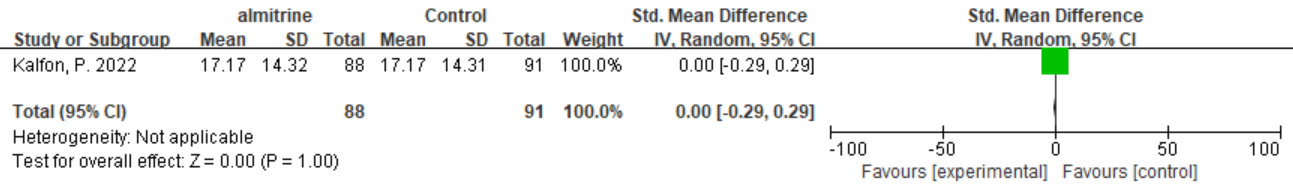  Figure S11 Hospital length of stay of almitrine for COVID-19(at the edge of sepsis)  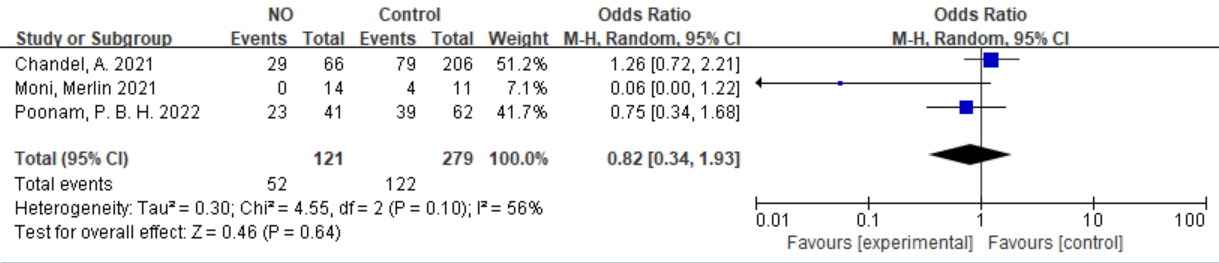  Figure S12 The need for intubation of inhalation NO for COVID-19(at the edge of sepsis)  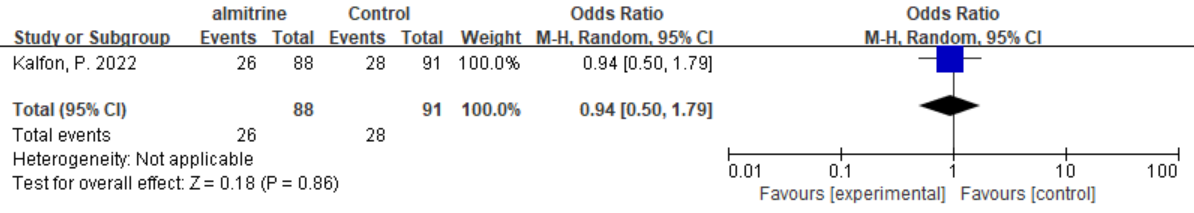  Figure S13 The need for intubation of almitrine for COVID-19(at the edge of sepsis)  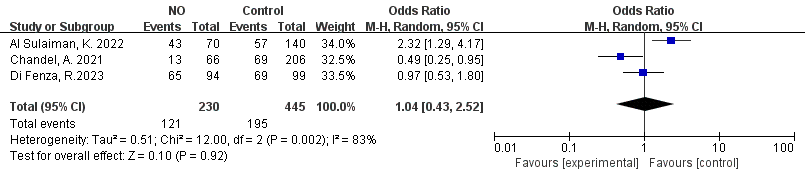  Figure S14 The SAEs of inhalation NO for COVID-19(at the edge of sepsis)  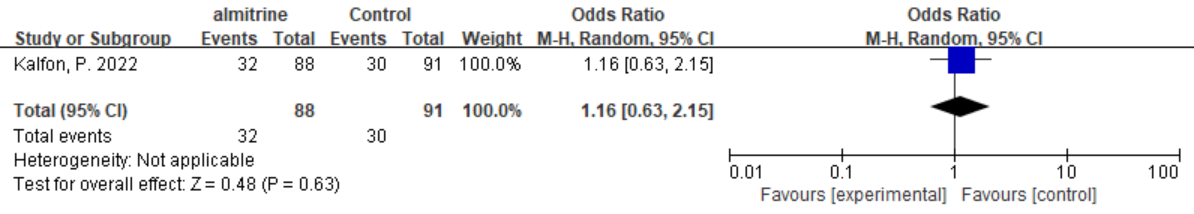  Figure S15 The SAEs of almitrine for COVID-19(at the edge of sepsis) | Supplementary 3 |
| Results of syntheses | 20a | The risk of bias of two RCTs were low to moderate, the results were showed in figure S1-2(Supplementary 3, appendix p1-2). The methodological quality of 15 cohorts were moderate to high, 1 case-control studies were moderate (NOS assessments results were showed in supplementary 3, appendix p3-19). The methodological quality of 3 case series were moderate, 7 case reports were moderate to high and one cross-sectional study were moderate (JBI assessments results were showed in supplementary 3, appendix, p20-30). | Results |
|  | 20b | Meta-analysis results showed that, inhaled NO did not affect the mortality (OR 0.96,95%CI 0.33-2.8, I^2^=81%, very low certainty), hospital length of stay (SMD 0.62, 95%CI 0.04-1.17, I2=83%, very low certainty), intubation needs (OR 0.82, 95%CI 0.34-1.93, I2=56%, very low certainty) and severe adverse events (SAEs)(OR 1.07, 95%CI 0.23-4.96,I2=92%, very low certainty) of patients with COVID-19(at the edge of sepsis). Meanwhile, almitrine did not affect the mortality (OR 0.44, 95%CI 0.17-1.13, low certainty), hospital length of stay (SMD 0.00, 95%CI -0.29-0.29, low certainty), intubation needs(OR 0.94, 95%CI 0.5-1.79, low certainty) and SAEs(OR 1.16, 95%CI 0.63-2.15, low certainty). Compared with pre-administration, the P/F of patients with NO(SMD-0.87, 95%CI -1.08--0.66,I2=0%, very low certainty), almitrine(SMD-0.73,95%CI-1.06--0.4,I2=1%, very low certainty), and NO combined with almitrine(SMD-0.94, 95%CI-1.71--0.16,I2=47%, very low certainty) increased significantly. | Results |
|  | 20c | Due to the lack of research, subgroup analysis and meta regression cannot be carried out. The doses of NO and almitrine in the included studies were not uniform, and differences in management schemes for patients with sepsis in different countries and additional variability during the COVID-19 pandemic would increase the heterogeneity of the findings. | Results |
|  | 20d | Due to lack of literature, sensitivity analysis cannot be carried out. | Results |
| Reporting biases | 21 | Due to lack of literature, sensitivity analysis cannot be carried out. | Results |
| Certainty of evidence | 22 | A summary of the GRADE assessment for certainty of evidence is provided in supplementary 3, appendix p35-38. | Results |
| **DISCUSSION** | | |  |
| Discussion | 23a | Meta-analysis results showed that, inhaled NO did not affect the mortality (OR 0.96,95%CI 0.33-2.8, I^2^=81%, very low certainty), hospital length of stay (SMD 0.62, 95%CI 0.04-1.17, I2=83%, very low certainty), intubation needs (OR 0.82, 95%CI 0.34-1.93, I2=56%, very low certainty) and severe adverse events (SAEs)(OR 1.07, 95%CI 0.23-4.96,I2=92%, very low certainty) of patients with COVID-19(at the edge of sepsis). Meanwhile, almitrine did not affect the mortality (OR 0.44, 95%CI 0.17-1.13, low certainty), hospital length of stay (SMD 0.00, 95%CI -0.29-0.29, low certainty), intubation needs(OR 0.94, 95%CI 0.5-1.79, low certainty) and SAEs(OR 1.16, 95%CI 0.63-2.15, low certainty). Compared with pre-administration, the P/F of patients with NO(SMD-0.87, 95%CI -1.08--0.66,I2=0%, very low certainty), almitrine(SMD-0.73,95%CI-1.06--0.4,I2=1%, very low certainty), and NO combined with almitrine(SMD-0.94, 95%CI-1.71--0.16,I2=47%, very low certainty) increased significantly. | Abstract |
|  | 23b | Firstly, some results of this study have significant heterogeneity, but due to the lack of research, subgroup analysis and meta regression cannot be carried out. However, despite the high heterogeneity of the research results, the research results can still reflect the trend of the efficacy of NO and almitrine. Meanwhile, the SOFA score or related indicators of some patients included in the study were median or mean, so we speculated that not all patients were septic patients, but the results of this population also reflected a trend problem. Because some patients might be or would be in a state of sepsis. | Discussion |
|  | 23c | Secondly, a lack of high-quality clinical researches limited our analyses. Majority of included studies were retrospective studies, these aspects could have introduced various confounders given the lack of risk adjustment or propensity score weighting. We included studies written only in English and Chinese, which also limits the scope of review. Thirdly, we found that SOFA scores of included patients varied, ethnic differences, the use of vasoactive drugs in many patients and prone position, and these factors did uncertain effects. Few studies to analyze the impact of these factors further and draw conclusions. Both ARDS and sepsis showed individual differences, which may also increase heterogeneity. | Discussion |
|  | 23d | Severe and critically ill patients admitted to the ICU due to COVID-19 are at higher risk of progressing to viral sepsis, and these patients would face more complex treatment. Data has shown that sepsis is one of the causes of death in patients with COVID-19 worldwide. Among the many vulnerable organs in sepsis patients, lung is the most vulnerable target organ, and patients often develop ARDS early, which is also one of the causes of death in sepsis patients. Therefore, sepsis and ARDS are not completely separated diseases in clinical treatment. At present, respiratory support is still the main treatment for sepsis and ARDS, with active removal of pathogens and symptomatic support. In addition to ARDS, sepsis may also show other organ dysfunction in clinic, such as coagulation function, liver and kidney function or central nervous system dysfunction. Overall, whether sepsis, ARDS, or COVID-19, there is an urgent need for more effective drugs. Unfortunately, medications for sepsis are limited and their efficacy and safety are controversial. Despite significant clinical and basic research efforts in sepsis (especially virus-associated sepsis), there are few effective drugs for this disease worldwide, and no definitive treatment recommendations have been made in authoritative guidelines. Considering the urgency of sepsis treatment during the COVID-19 pandemic, people are trying to screen out drugs with potential therapeutic value from previous treatments.  Significant vascular endothelial injury and a higher incidence of pulmonary microthrombus were observed in critically ill patients with COVID-19, leading to poor oxygenation and pulmonary changes. Most critically ill patients require mechanical ventilation due to difficulties in maintaining oxygenation and ventilation, which remains a major challenge for critically ill patients with COVID-19. During the COVID-19 pandemic, it is essential to increase the number of days without ventilators for critically ill patients and minimize the need for respiratory support equipment. Therefore, dilating smooth muscle vessels and increasing alveolar blood flow to enhance oxygenation may be an option for treating critical illness. NO induces relaxation of vascular smooth muscle and dilates pulmonary blood vessels, thereby improving blood oxygenation and reducing right-to-left shunt in the lung. Almitrine reduces intrapulmonary shunt by enhancing hypoxic pulmonary vasoconstriction, which has been reported in patients with severe hypoxemia. NO and almitrine have been reported as a rescue strategy for 'classical' ARDS in patients with severe hypoxemia. This treatment increased the P/F and reduced physiologic dead space fraction over 24 hours, but did not improve mortality or length of ICU stay. Almitrine and inhalation of NO was considered by some experts as a salvage treatment strategy for critically ill patients during the COVID-19 pandemic, including refractory hypoxaemia. However, these treatments remain controversy. As we all known, COVID-19 patients with SOFA score ≥ 2, who were already in the state of sepsis or were about to determine into sepsis, and these patients urgently needed appropriate, safe and effective treatment. In this study, we evaluated the efficacy and safety of NO, almitrine, and NO-almitrine combination to provide reliable and up-to-date clinical evidence and research ideas for treatment. | Discussion |
| **OTHER INFORMATION** | | |  |
| Registration and protocol | 24a | PROSPERO registration number: CRD42022367667 | Methods |
|  | 24b | Available from: https://wwwcrdyorkacuk/prospero/display_recordphp?ID=CRD42022367667 2022. | Reference |
|  | 24c | Not modified. | PROSPERO website |
| Support | 25 | This research did not receive any specific grant from funding agencies in the public, commercial, or not-for-profit sectors. | Manuscript |
| Competing interests | 26 | The authors declare that the research was conducted in the absence of any commercial or financial relationships that could be construed as a potential conflict of interest. | Manuscript |
| Availability of data, code and other materials | 27 | Any other materials used in the review. | Supplementary 1-2 |

*From:*  Page MJ, McKenzie JE, Bossuyt PM, Boutron I, Hoffmann TC, Mulrow CD, et al. The PRISMA 2020 statement: an updated guideline for reporting systematic reviews. BMJ 2021;372:n71. doi: 10.1136/bmj.n71

**Supplementary 1 table 2. Search strategy**

| **PubMed search strategy – last searched on Oct 23, 2023** | | **Results** |
| --- | --- | --- |
| #1 | Search: "Nitric Oxide"[MeSH Terms] OR ("nitric"[All Fields] AND "oxide"[All Fields]) OR "Nitric Oxide"[All Fields] OR (("administration, inhalation"[MeSH Terms] OR ("administration"[All Fields] AND "inhalation"[All Fields]) OR "inhalation administration"[All Fields] OR "inhalant"[All Fields] OR "inhalability"[All Fields] OR "inhalable"[All Fields] OR "inhalants"[All Fields] OR "inhalated"[All Fields] OR "inhalation"[MeSH Terms] OR "inhalation"[All Fields] OR "inhal"[All Fields] OR "inhalations"[All Fields] OR "inhale"[All Fields] OR "inhaled"[All Fields] OR "inhaling"[All Fields] OR "inhalational"[All Fields] OR "inhalative"[All Fields] OR "inhalatively"[All Fields] OR "inhalent"[All Fields] OR "inhaler s"[All Fields] OR "inhales"[All Fields] OR "nebulizers and vaporizers"[MeSH Terms] OR ("nebulizers"[All Fields] AND "vaporizers"[All Fields]) OR "nebulizers and vaporizers"[All Fields] OR "inhalator"[All Fields] OR "inhalators"[All Fields] OR "inhaler"[All Fields] OR "inhalers"[All Fields]) AND ("Nitric Oxide"[MeSH Terms] OR ("nitric"[All Fields] AND "oxide"[All Fields]) OR "Nitric Oxide"[All Fields])) OR ("administration, inhalation"[MeSH Terms] OR ("administration"[All Fields] AND "inhalation"[All Fields]) OR "inhalation administration"[All Fields] OR "inhalant"[All Fields] OR "inhalability"[All Fields] OR "inhalable"[All Fields] OR "inhalants"[All Fields] OR "inhalated"[All Fields] OR "inhalation"[MeSH Terms] OR "inhalation"[All Fields] OR "inhal"[All Fields] OR "inhalations"[All Fields] OR "inhale"[All Fields] OR "inhaled"[All Fields] OR "inhaling"[All Fields] OR "inhalational"[All Fields] OR "inhalative"[All Fields] OR "inhalatively"[All Fields] OR "inhalent"[All Fields] OR "inhaler s"[All Fields] OR "inhales"[All Fields] OR "nebulizers and vaporizers"[MeSH Terms] OR ("nebulizers"[All Fields] AND "vaporizers"[All Fields]) OR "nebulizers and vaporizers"[All Fields] OR "inhalator"[All Fields] OR "inhalators"[All Fields] OR "inhaler"[All Fields] OR "inhalers"[All Fields]) OR "iNO"[All Fields] OR ("Nitric Oxide"[MeSH Terms] OR "Nitric Oxide Synthase Type III"[MeSH Terms] OR "Nitric Oxide Synthase Type I"[MeSH Terms] OR "Nitric Oxide Synthase Type II"[MeSH Terms] OR "Nitric Oxide Donors"[MeSH Terms] OR "Nitric Oxide Synthase"[MeSH Terms] OR "nos2 protein human"[Supplementary Concept] OR "Nitric Oxide Donors"[Pharmacological Action]) | 388,466 |
| #2 | Search: "severe acute respiratory syndrome coronavirus 2"[Title/Abstract] OR "severe acute respiratory syndrome corona virus 2"[Title/Abstract] OR "SARS-COV-2"[Title/Abstract] OR "SARS 2"[Title/Abstract] | 131,203 |
| #3 | Search: "coronavirus disease 2019"[Title/Abstract] OR "coronavirus disease-19"[Title/Abstract] OR "COVID-19"[Title/Abstract] OR "COVID19"[Title/Abstract] OR "2019 coronavirus"[Title/Abstract] OR "2019 novel coronavirus"[Title/Abstract] OR "2019-nCoV"[Title/Abstract] OR "Novel coronavirus pneumonia"[Title/Abstract] OR "NCP"[Title/Abstract] OR "coronavirus disease"[Title/Abstract] OR "corona virus disease 2019"[Title/Abstract] OR "coronavirus 2019"[Title/Abstract] OR "human coronavirus 2019"[Title/Abstract] OR "HCoV-19"[Title/Abstract] | 350,874 |
| #4 | Search: "covid 19 virus variants"[Title/Abstract] OR "SARS-CoV-2 variants"[Title/Abstract] OR "SARS-CoV-2 variants"[Supplementary Concept] | 9,664 |
| #5 | Search: #2 OR #3 OR #4 | 377,270 |
| #6 | Search: "Almitrine"[MeSH Terms] OR "Almitrine"[All Fields] OR "Almitrine"[MeSH Terms] OR "almitrine raubasine drug combination"[Supplementary Concept] | 582 |
| #7 | Search: #1 AND #5 | 2,928 |
| #8 | Search: #5 AND #6 | 18 |

**Supplementary 1 table 3. Search strategy**

| **Cochrane Library search strategy – last searched on Oct 23, 2023** | | **Results** |
| --- | --- | --- |
| #1 | (nitric oxide):ti,ab,kw OR (NO):ti,ab,kw OR (nitric oxide donor):ti,ab,kw OR (inhaled nitric oxide):ti,ab,kw | 8642 |
| #2 | MeSH descriptor: [nitric oxide] explode all trees | 197 |
| #3 | (almitrine):ti,ab,kw OR (almitrine raubasine drug combination):ti,ab,kw | 214 |
| #4 | (2019 nCoV):ti,ab,kw OR (COVID-19):ti,ab,kw OR (COVID-19 Virus Disease):ti,ab,kw OR (SARS-CoV-2):ti,ab,kw OR (coronavirus):ti,ab,kw | 3,978 |
| #5 | #3 AND #4 | 4 |
| #6 | (#1 OR #2) AND #4 | 95 |

**Supplementary 1 table 4. Search strategy**

| **Embase search strategy – last searched on Oct 23, 2023** | | **Results** |
| --- | --- | --- |
| #1 | 'nitric oxide'/exp OR 'nitric oxide':ti,ab,kw OR genosyl:ti,ab,kw OR inomax:ti,ab,kw OR no:ti,ab,kw OR 'nitrogen monoxide':ti,ab,kw | 7,154,292 |
| #2 | ‘severe acute respiratory syndrome coronavirus 2’/exp OR ‘covid 19’/exp OR ‘coronavirus disease 2019’/exp OR ((2019 OR 19 OR wuhan) NEAR/10 (covid OR coronavirus OR nCoV OR “corona virus” OR CoV)):ti,ab OR SARSCoV-2:ti,ab | 448,850 |
| #3 | ‘almitrine‘/exp OR almitrine:ti,ab,kw OR (1:ti,ab,kw AND '4, 6 diallylamino 2 triazinyl':ti,ab,kw AND 4:ti,ab,kw AND '4, 4 difluorobenzhydryl':ti,ab,kw AND piperazine:ti,ab,kw) OR ('1 [4, 6 bis':ti,ab,kw AND allylamino:ti,ab,kw AND 's triazin 2 yl] 4':ti,ab,kw AND '4, 4 difluorobenzhydryl':ti,ab,kw AND piperazine:ti,ab,kw) OR ('2, 4 bis':ti,ab,kw AND allylamino:ti,ab,kw AND '6 [4 [bis':ti,ab,kw AND '4 fluorophenyl':ti,ab,kw AND 'methyl] 1 piperazinyl] 1, 3, 5 triazine':ti,ab,kw) OR ('2, 4 bis':ti,ab,kw AND allylamino:ti,ab,kw AND '6 [4 [bis':ti,ab,kw AND '4 fluorophenyl':ti,ab,kw AND 'methyl] piperazin 1 yl] s triazine; almitrine bismethane sulfonate':ti,ab,kw) OR ('n, n diallyl 6 [4':ti,ab,kw AND '4, 4 difluorobenzhydryl':ti,ab,kw AND 'piperazin 1 yl] 1, 3, 5 triazine 2, 4 diyldiamine':ti,ab,kw) | 1,122 |
| #4 | #1 AND #2 | 80,001 |
| #5 | #2 AND #3 | 44 |

**Supplementary 1 table 5. List of** **excluded studies**

| **Author (Reference)** | **Reason for exclusion** |
| --- | --- |
| Alvarez, R. A.[1] | Review |
| Chandel, A.[2] | Meeting Abstract |
| Easterlin, M. C.[3] | The dose of nitric oxide was not reported. |
| Firstenberg, M. S.[4] | No enough information about post-administration of nitric oxide. |
| Joshi, A. Y.[5] | No enough information to identify patients as sepsis. |
| Matthews, L.[6] | No enough information to separate nitric oxide group. |
| Piecek, J.[7] | No enough information to separate COVID-19 group. |
| Safaee Fakhr, B.[8] | No enough information to identify patients as sepsis. |
| Srivastava, S.[9] | Short communication |
| Strickland, B.[10] | No enough information to identify patients as sepsis. |
| Tandon, M.[11] | No enough information to identify patients as sepsis. |
| Valsecchi, C.[12] | No enough information to identify patients as sepsis. |
| Wiegand, S. B.[13] | No enough information to identify patients as sepsis. |
| Winchester, S.[14] | No enough information to identify patients as sepsis. |
| Zamanian, R. T.[15] | No enough information to identify patients as sepsis. |
| D'Souza, R.[16] | Review |
| Ogulur, I.[17] | Review |
| Fang, W.[18] | Review |
| Kamenshchikov, N. O.[19] | Review |
| Martel, J.[20] | Review |
| Jani, V. P.[21] | Review |
| Qin, X.[22] | No enough information to identify patients as sepsis. |
| Mir, J. M.[23] | Review |
| Adusumilli, N. C.[24] | Review |
| Pieretti, J. C.[25] | Review |
| Bobot, M.[26] | No enough information to separate nitric oxide group. |
| Lei, C.[27] | No enough information to identify patients as sepsis. |
| Udrea, D. S.[28] | No enough information to identify patients as sepsis. |
| Patel, P. A.[29] | No enough information to identify patients as sepsis. |
| Pozdnyakov, A.[30] | No enough information about post-administration of nitric oxide. |
| Guimarães, L. M. F.[31] | Review |
| Escribano-Santana, I.[32] | Review |
| Robba, C.[33] | No enough information to separate nitric oxide group. |
| Dal Moro, F.[34] | Review |
| Matera, M. G.[35] | Review |
| Karlsson, J. O. G.[36] | No enough information to identify patients as sepsis. |
| Jozwiak, M.[37] | No enough information to separate nitric oxide group. |
| Donoso Fuentes, A.[38] | Spanish |
| Price, L. C.[39] | No enough information to separate nitric oxide group. |
| Osho, A. A.[40] | No enough information about post-administration of nitric oxide. |
| Derespina, K. R.[41] | No enough information to separate nitric oxide group. |
| Pinciroli, R.[42] | No enough information to identify patients as sepsis. |
| Hamid, K.[43] | No enough information to identify patients as COVID-19. |
| Ricciardolo, F. L. M.[44] | Review |
| Frostell, C. G.[45] | No enough information to identify patients as sepsis. |
| Shei, R. J.[46] | Review |
| Bendjelid, K.[47] | Editorial |

**References of excluded studies**

1. Alvarez RA, Berra L, Gladwin MT: Home Nitric Oxide Therapy for COVID-19. *American journal of respiratory and critical care medicine* 2020, 202(1):16-20.

2. Chandel A, Patolia S, Ahmad K, Aryal S, Brown AW, Sahjwani D, Khangoora V, Shlobin OA, Cameron PC, Singhal A *et al*: Inhaled Nitric Oxide via High-Flow Nasal Cannula in Patients with Acute Respiratory Failure Related to COVID-19. *Clinical medicine insights Circulatory, respiratory and pulmonary medicine* 2021, 15:11795484211047065.

3. Easterlin MC, De Beritto T, Yeh AM, Wertheimer FB, Ramanathan R: Extremely Preterm Infant Born to a Mother With Severe COVID-19 Pneumonia. *J Investig Med High Impact Case Rep* 2020, 8:2324709620946621.

4. Firstenberg MS, Stahel PF, Hanna J, Kotaru C, Crossno J, Jr., Forrester J: Successful COVID-19 rescue therapy by extra-corporeal membrane oxygenation (ECMO) for respiratory failure: a case report. *Patient safety in surgery* 2020, 14:20.

5. Joshi AY, Mullakary RM, Iyer VN: Successful treatment of coronavirus disease 2019 in a patient with asthma. *Allergy and asthma proceedings* 2020, 41(4):296-300.

6. Matthews L, Baker L, Ferrari M, Sanchez W, Pappachan J, Grocott MP, Dushianthan A: Compassionate use of Pulmonary Vasodilators in Acute Severe Hypoxic Respiratory Failure due to COVID-19. *Journal of intensive care medicine* 2022, 37(8):1101-1111.

7. Piecek J, Valentino T, Aust R, Harris L, Hancock J, Hardman C, van Poppel SF: The Use of Nitric Oxide as a Rescue Modality for Severe Adult Acute Respiratory Distress Syndrome Patients, Including COVID-19, in Critical Care Rotor Transport: A Retrospective Community Outcome Study. *Air medical journal* 2022, 41(5):427-431.

8. Safaee Fakhr B, Di Fenza R, Gianni S, Wiegand SB, Miyazaki Y, Araujo Morais CC, Gibson LE, Chang MG, Mueller AL, Rodriguez-Lopez JM *et al*: Inhaled high dose nitric oxide is a safe and effective respiratory treatment in spontaneous breathing hospitalized patients with COVID-19 pneumonia. *Nitric oxide : biology and chemistry* 2021, 116:7-13.

9. Srivastava S, Garg I, Hembrom AA, Kumar B: Assessment of nitric oxide (NO) potential to mitigate COVID-19 severity. *Virusdisease* 2021, 32(3):589-594.

10. Strickland B, Albala L, Coffey EC, Carroll RW, Zapol WM, Ichinose F, Berra L, Harris NS: Safety and practicality of high dose inhaled nitric oxide in emergency department COVID-19 patients. *The American journal of emergency medicine* 2022, 58:5-8.

11. Tandon M, Wu W, Moore K, Winchester S, Tu YP, Miller C, Kodgule R, Pendse A, Rangwala S, Joshi S: SARS-CoV-2 accelerated clearance using a novel nitric oxide nasal spray (NONS) treatment: A randomized trial. *The Lancet regional health Southeast Asia* 2022, 3:100036.

12. Valsecchi C, Winterton D, Safaee Fakhr B, Collier AY, Nozari A, Ortoleva J, Mukerji S, Gibson LE, Carroll RW, Shaefi S *et al*: High-Dose Inhaled Nitric Oxide for the Treatment of Spontaneously Breathing Pregnant Patients With Severe Coronavirus Disease 2019 (COVID-19) Pneumonia. *Obstetrics and gynecology* 2022, 140(2):195-203.

13. Wiegand SB, Safaee Fakhr B, Carroll RW, Zapol WM, Kacmarek RM, Berra L: Rescue Treatment With High-Dose Gaseous Nitric Oxide in Spontaneously Breathing Patients With Severe Coronavirus Disease 2019. *Critical care explorations* 2020, 2(11):e0277.

14. Winchester S, John S, Jabbar K, John I: Clinical efficacy of nitric oxide nasal spray (NONS) for the treatment of mild COVID-19 infection. *The Journal of infection* 2021, 83(2):237-279.

15. Zamanian RT, Pollack CV, Jr., Gentile MA, Rashid M, Fox JC, Mahaffey KW, de Jesus Perez V: Outpatient Inhaled Nitric Oxide in a Patient with Vasoreactive Idiopathic Pulmonary Arterial Hypertension and COVID-19 Infection. *American journal of respiratory and critical care medicine* 2020, 202(1):130-132.

16. D'Souza R, Ashraf R, Rowe H, Zipursky J, Clarfield L, Maxwell C, Arzola C, Lapinsky S, Paquette K, Murthy S *et al*: Pregnancy and COVID-19: pharmacologic considerations. *Ultrasound in obstetrics & gynecology : the official journal of the International Society of Ultrasound in Obstetrics and Gynecology* 2021, 57(2):195-203.

17. Ogulur I, Pat Y, Ardicli O, Barletta E, Cevhertas L, Fernandez-Santamaria R, Huang M, Bel Imam M, Koch J, Ma S *et al*: Advances and highlights in biomarkers of allergic diseases. *Allergy* 2021, 76(12):3659-3686.

18. Fang W, Jiang J, Su L, Shu T, Liu H, Lai S, Ghiladi RA, Wang J: The role of NO in COVID-19 and potential therapeutic strategies. *Free radical biology & medicine* 2021, 163:153-162.

19. Kamenshchikov NO, Berra L, Carroll RW: Therapeutic Effects of Inhaled Nitric Oxide Therapy in COVID-19 Patients. *Biomedicines* 2022, 10(2).

20. Martel J, Ko YF, Young JD, Ojcius DM: Could nasal nitric oxide help to mitigate the severity of COVID-19? *Microbes and infection* 2020, 22(4-5):168-171.

21. Jani VP, Munoz CJ, Govender K, Williams AT, Cabrales P: Implications of microvascular dysfunction and nitric oxide mediated inflammation in severe COVID-19 infection. *The American journal of the medical sciences* 2022, 364(3):251-256.

22. Qin X, Huang C, Wu K, Li Y, Liang X, Su M, Li R: Anti-coronavirus disease 2019 (COVID-19) targets and mechanisms of puerarin. *Journal of cellular and molecular medicine* 2021, 25(2):677-685.

23. Mir JM, Maurya RC: Nitric oxide boosters as defensive agents against COVID-19 infection: an opinion. *Journal of biomolecular structure & dynamics* 2022, 40(9):4285-4291.

24. Adusumilli NC, Zhang D, Friedman JM, Friedman AJ: Harnessing nitric oxide for preventing, limiting and treating the severe pulmonary consequences of COVID-19. *Nitric oxide : biology and chemistry* 2020, 103:4-8.

25. Pieretti JC, Rubilar O, Weller RB, Tortella GR, Seabra AB: Nitric oxide (NO) and nanoparticles - Potential small tools for the war against COVID-19 and other human coronavirus infections. *Virus research* 2021, 291:198202.

26. Bobot M, Tonon D, Peres N, Guervilly C, Lefèvre F, Max H, Bommel Y, Volff M, Leone M, Lopez A *et al*: Impact of Dexamethasone and Inhaled Nitric Oxide on Severe Acute Kidney Injury in Critically Ill Patients with COVID-19. *J Clin Med* 2022, 11(20).

27. Lei C, Su B, Dong H, Bellavia A, Di Fenza R, Safaee Fakhr B, Gianni S, Grassi LG, Kacmarek R, Araujo Morais CC *et al*: Protocol of a randomized controlled trial testing inhaled Nitric Oxide in mechanically ventilated patients with severe acute respiratory syndrome in COVID-19 (SARS-CoV-2). *medRxiv : the preprint server for health sciences* 2020.

28. Udrea DS, Lopez M, Avesar M, Qureshi S, Moretti A, Abd-Allah SA, Chandnani HK: Acute COVID-19 Infection in a Pediatric Patient with ROHHAD. *Journal of pediatric genetics* 2022, 11(4):309-312.

29. Patel PA, Chandrakasan S, Mickells GE, Yildirim I, Kao CM, Bennett CM: Severe Pediatric COVID-19 Presenting With Respiratory Failure and Severe Thrombocytopenia. *Pediatrics* 2020, 146(1).

30. Pozdnyakov A, Jin A, Bader M: Reactivation of Pulmonary Tuberculosis in a Patient With COVID-19: Case Report and Review of Literature. *Infectious diseases in clinical practice (Baltimore, Md)* 2021, 29(6):e468-e470.

31. Guimarães LMF, Rossini CVT, Lameu C: Implications of SARS-Cov-2 infection on eNOS and iNOS activity: Consequences for the respiratory and vascular systems. *Nitric oxide : biology and chemistry* 2021, 111-112:64-71.

32. Escribano-Santana I, Martínez-Gimeno ML, Herráiz-Bermejo L: [Adjuvant Treatments to Invasive Mechanical Ventilation in the management of Acute Respiratory Distress Syndrome secondary to Covid-19]. *Enfermeria intensiva* 2022.

33. Robba C, Ball L, Battaglini D, Cardim D, Moncalvo E, Brunetti I, Bassetti M, Giacobbe DR, Vena A, Patroniti N *et al*: Early effects of ventilatory rescue therapies on systemic and cerebral oxygenation in mechanically ventilated COVID-19 patients with acute respiratory distress syndrome: a prospective observational study. *Critical care (London, England)* 2021, 25(1):111.

34. Dal Moro F, Vendramin I, Livi U: The war against the SARS-CoV2 infection: Is it better to fight or mitigate it? *Medical hypotheses* 2020, 143:110129.

35. Matera MG, Imperatore F, Annibale R, Cazzola M: Advances in the Pharmacological Management of Pediatric Acute Respiratory Distress Syndrome. *Expert Opin Pharmacother* 2022, 23(3):349-360.

36. Karlsson JOG, Jynge P, Ignarro LJ: May Mangafodipir or Other SOD Mimetics Contribute to Better Care in COVID-19 Patients? *Antioxidants (Basel, Switzerland)* 2020, 9(10).

37. Jozwiak M, Chiche JD, Charpentier J, Ait Hamou Z, Jaubert P, Benghanem S, Dupland P, Gavaud A, Péne F, Cariou A *et al*: Use of Venovenous Extracorporeal Membrane Oxygenation in Critically-Ill Patients With COVID-19. *Frontiers in medicine* 2020, 7:614569.

38. Donoso Fuentes A, Diaz Rubio F: [Inhaled nitric oxide and prone position as a rescue therapy for severe hypoxemia in an infant with COVID-19 pneumonia]. *Andes pediatrica : revista Chilena de pediatria* 2021, 92(3):483-484.

39. Price LC, Garfield B, Bleakley C, Keeling AGM, McFadyen C, McCabe C, Ridge CA, Wort SJ, Price S, Arachchillage DJ: Rescue therapy with thrombolysis in patients with severe COVID-19-associated acute respiratory distress syndrome. *Pulmonary circulation* 2020, 10(4):2045894020973906.

40. Osho AA, Moonsamy P, Hibbert KA, Shelton KT, Trahanas JM, Attia RQ, Bloom JP, Onwugbufor MT, D'Alessandro DA, Villavicencio MA *et al*: Veno-venous Extracorporeal Membrane Oxygenation for Respiratory Failure in COVID-19 Patients: Early Experience From a Major Academic Medical Center in North America. *Annals of surgery* 2020, 272(2):e75-e78.

41. Derespina KR, Kaushik S, Plichta A, Conway EE, Jr., Bercow A, Choi J, Eisenberg R, Gillen J, Sen AI, Hennigan CM *et al*: Clinical Manifestations and Outcomes of Critically Ill Children and Adolescents with Coronavirus Disease 2019 in New York City. *The Journal of pediatrics* 2020, 226:55-63.e52.

42. Pinciroli R, Traeger L, Fischbach A, Gianni S, Morais CCA, Fakhr BS, Di Fenza R, Robinson D, Carroll R, Zapol WM *et al*: A Novel Inhalation Mask System to Deliver High Concentrations of Nitric Oxide Gas in Spontaneously Breathing Subjects. *Journal of visualized experiments : JoVE* 2021(171).

43. Hamid K, Ali M, Devasahayam J: Acute Respiratory Distress Syndrome Secondary to Enterovirus-Human-Rhinovirus Infection in an Adult. *Cureus* 2022, 14(6):e26475.

44. Ricciardolo FLM, Bertolini F, Carriero V, Högman M: Nitric oxide's physiologic effects and potential as a therapeutic agent against COVID-19. *Journal of breath research* 2020, 15(1):014001.

45. Frostell CG, Hedenstierna G: Nitric oxide and COVID-19: Dose, timing and how to administer it might be crucial. *Acta anaesthesiologica Scandinavica* 2021, 65(5):576-577.

46. Shei RJ, Baranauskas MN: More questions than answers for the use of inhaled nitric oxide in COVID-19. *Nitric oxide : biology and chemistry* 2022, 124:39-48.

47. Bendjelid K, Giraud R, Von Düring S: Treating hypoxemic COVID-19 "ARDS" patients with almitrine: The earlier the better? *Anaesthesia, critical care & pain medicine* 2020, 39(4):451-452.

**References of included studies**

1. Al Sulaiman K, Korayem GB, Altebainawi AF, Al Harbi S, Alissa A, Alharthi A, Kensara R, Alfahed A, Vishwakarma R, Al Haji H *et al*: Evaluation of inhaled nitric oxide (iNO) treatment for moderate-to-severe ARDS in critically ill patients with COVID-19: a multicenter cohort study. *Critical care (London, England)* 2022, 26(1):304.
2. Giri AR, Yarrarapu SNS, Kaur N, Hochwald A, Crook J, Helgeson S, Harrison MF, Patel N, Guru PK, Lowman P *et al*: Inhaled nitric oxide use in COVID19-induced hypoxemic respiratory failure. 2021:2021.2008.2019.21262314.
3. 19. Moni M, Madathil T, Sathyapalan DT, Menon V, Gutjahr G, Edathadathil F, Sureshkumar D, Prasanna P, Jose S, Jerome R *et al*: A Feasibility Trial to Evaluate the Composite Efficacy of Inhaled Nitric Oxide in the Treatment of Covid 19 Pneumonia : Impact on Viral Load and Clinical Outcomes. 2021:2021.2004.2015.21255300.
4. 20. Lotz C, Muellenbach RM, Meybohm P, Mutlak H, Lepper PM, Rolfes CB, Peivandi A, Stumpner J, Kredel M, Kranke P *et al*: Effects of inhaled nitric oxide in COVID-19-induced ARDS - Is it worthwhile? *Acta anaesthesiologica Scandinavica* 2021, 65(5):629-632.
5. 21. Brown CJ, Rubel N, Lai J, Ward C, McLean J, Wheelock M, Steuerwald M, Cathers A: Initiation of Inhaled Nitric Oxide by an Air Transport Team in Adult Coronavirus Disease 2019 Respiratory Failure. *Air medical journal* 2022, 41(4):406-410.
6. 22. Vives M, Gascó I, Pla G, Maciel JL, Ricart Hernandez A, Regí Roman K, Parramon F: Inhaled Nitric Oxide in Acute Severe Pulmonary Hypertension and Severe Acute Respiratory Distress Syndrome Secondary to COVID-19 Pneumonia: A Case Report. *The American journal of case reports* 2022, 23:e937147.
7. 23. Cardinale M, Esnault P, Cotte J, Cungi PJ, Goutorbe P: Effect of almitrine bismesylate and inhaled nitric oxide on oxygenation in COVID-19 acute respiratory distress syndrome. *Anaesthesia, critical care & pain medicine* 2020, 39(4):471-472.
8. 24. Losser MR, Lapoix C, Delannoy M, Champigneulle B, Payen D: Almitrine as a non-ventilatory strategy to improve intrapulmonary shunt in COVID-19 patients. *Anaesthesia, critical care & pain medicine* 2020, 39(4):467-469.
9. 25. Bagate F, Tuffet S, Masi P, Perier F, Razazi K, de Prost N, Carteaux G, Payen D, Mekontso Dessap A: Rescue therapy with inhaled nitric oxide and almitrine in COVID-19 patients with severe acute respiratory distress syndrome. *Annals of intensive care* 2020, 10(1):151.
10. 26. Garfield B, McFadyen C, Briar C, Bleakley C, Vlachou A, Baldwin M, Lees N, Price S, Ledot S, McCabe C *et al*: Potential for personalised application of inhaled nitric oxide in COVID-19 pneumonia. *British journal of anaesthesia* 2021, 126(2):e72-e75.
11. 27. Longobardo A, Montanari C, Shulman R, Benhalim S, Singer M, Arulkumaran N: Inhaled nitric oxide minimally improves oxygenation in COVID-19 related acute respiratory distress syndrome. *British journal of anaesthesia* 2021, 126(1):e44-e46.
12. 28. Paramanathan S, Kyng KJ, Laursen AL, Jensen LD, Grejs AM, Jain D: COVID-19 with severe acute respiratory distress in a pregnant woman leading to preterm caesarean section: A case report. *Case reports in women's health* 2021, 30:e00304.
13. 29. Barthélémy R, Blot PL, Tiepolo A, Le Gall A, Mayeur C, Gaugain S, Morisson L, Gayat E, Mebazaa A, Chousterman BG: Efficacy of Almitrine in the Treatment of Hypoxemia in Sars-Cov-2 Acute Respiratory Distress Syndrome. *Chest* 2020, 158(5):2003-2006.
14. 30. Chandel A, Patolia S, Ahmad K, Aryal S, Brown AW, Sahjwani D, Khangoora V, Shlobin OA, Cameron PC, Singhal A *et al*: Inhaled Nitric Oxide via High-Flow Nasal Cannula in Patients with Acute Respiratory Failure Related to COVID-19. *Clinical medicine insights Circulatory, respiratory and pulmonary medicine* 2021, 15:11795484211047065.
15. 31. Abou-Arab O, Huette P, Debouvries F, Dupont H, Jounieaux V, Mahjoub Y: Inhaled nitric oxide for critically ill Covid-19 patients: a prospective study. *Critical care (London, England)* 2020, 24(1):645.
16. 32. Tavazzi G, Pozzi M, Mongodi S, Dammassa V, Romito G, Mojoli F: Inhaled nitric oxide in patients admitted to intensive care unit with COVID-19 pneumonia. *Critical care (London, England)* 2020, 24(1):508.
17. 33. Ziehr DR, Alladina J, Wolf ME, Brait KL, Malhotra A, La Vita C, Berra L, Hibbert KA, Hardin CC: Respiratory Physiology of Prone Positioning With and Without Inhaled Nitric Oxide Across the Coronavirus Disease 2019 Acute Respiratory Distress Syndrome Severity Spectrum. *Critical care explorations* 2021, 3(6):e0471.
18. 34. Caplan M, Goutay J, Bignon A, Jaillette E, Favory R, Mathieu D, Parmentier-Decrucq E, Poissy J, Duburcq T: Almitrine Infusion in Severe Acute Respiratory Syndrome Coronavirus 2-Induced Acute Respiratory Distress Syndrome: A Single-Center Observational Study. *Critical care medicine* 2021, 49(2):e191-e198.
19. 35. Kalfon P, Payen JF, Rousseau A, Chousterman B, Cachanado M, Tibi A, Audibert J, Depret F, Constantin JM, Weiss E *et al*: Effect of intravenous almitrine on intubation or mortality in patients with COVID-19 acute hypoxemic respiratory failure: A multicentre, randomised, double-blind, placebo-controlled trial. *EClinicalMedicine* 2022, 52:101663.
20. 36. Feng WX, Yang Y, Wen J, Liu YX, Liu L, Feng C: Implication of inhaled nitric oxide for the treatment of critically ill COVID-19 patients with pulmonary hypertension. *ESC heart failure* 2021, 8(1):714-718.
21. 37. Laghlam D, Rahoual G, Malvy J, Estagnasié P, Brusset A, Squara P: Use of Almitrine and Inhaled Nitric Oxide in ARDS Due to COVID-19. *Frontiers in medicine* 2021, 8:655763.
22. 38. Heuts S, Ubben JF, Banks-Gonzales V, Sels JW, Lorusso R, van Mook W, Delnoij TSR: Nitric Oxide Ventilation Improves Recirculation and Right Ventricular Function During Veno-Venous Extracorporeal Membrane Oxygenation in a COVID-19 Patient. *Journal of cardiothoracic and vascular anesthesia* 2021, 35(9):2763-2767.
23. 39. Ferrari M, Santini A, Protti A, Andreis DT, Iapichino G, Castellani G, Rendiniello V, Costantini E, Cecconi M: Inhaled nitric oxide in mechanically ventilated patients with COVID-19. *Journal of critical care* 2020, 60:159-160.
24. 40. Lubinsky AS, Brosnahan SB, Lehr A, Elnadoury O, Hagedorn J, Garimella B, Bender MT, Amoroso N, Artigas A, Bos LDJ *et al*: Inhaled pulmonary vasodilators are not associated with improved gas exchange in mechanically ventilated patients with COVID-19: A retrospective cohort study. *Journal of critical care* 2022, 69:153990.
25. 41. Herranz L, da Silveira JG, Trocado LFL, Alvaraes AL, Fittipaldi J: Inhaled Nitric Oxide in Patients with Severe COVID-19 Infection at Intensive Care Unit - A Cross Sectional Study. *Journal of critical care medicine (Universitatea de Medicina si Farmacie din Targu-Mures)* 2021, 7(4):318-319.
26. 42. Huette P, Beyls C, Guilbart M, Haye G, Najid FZ, Mestan B, Roger PA, Dupont H, Abou-Arab O, Mahjoub Y: Acute Cor Pulmonale in COVID-19-Related ARDS: Improvement With Almitrine Infusion. *JACC Case reports* 2020, 2(9):1311-1314.
27. 43. Safaee Fakhr B, Wiegand SB, Pinciroli R, Gianni S, Morais CCA, Ikeda T, Miyazaki Y, Marutani E, Di Fenza R, Larson GM *et al*: High Concentrations of Nitric Oxide Inhalation Therapy in Pregnant Patients With Severe Coronavirus Disease 2019 (COVID-19). *Obstetrics and gynecology* 2020, 136(6):1109-1113.
28. 44. Poonam PBH, Koscik R, Nguyen T, Rikhi S, Lin HM: Nitric oxide versus epoprostenol for refractory hypoxemia in Covid-19. *PLoS One* 2022, 17(6):e0270646.
29. 45. Huette P, Abou Arab O, Jounieaux V, Guilbart M, Belhout M, Haye G, Dupont H, Beyls C, Mahjoub Y: Almitrine for COVID-19 critically ill patients - a vascular therapy for a pulmonary vascular disease: Three case reports. *World journal of clinical cases* 2021, 9(14):3385-3393.
30. 46. Saccheri C, Morand L, Juston M, Doyen D, Hyvernat H, Lombardi R, Devanlay R, Panicucci É, Dellamonica J, Jozwiak M: Use of almitrine in spontaneously breathing patients with COVID-19 treated with high-flow nasal cannula oxygen therapy and with persistent hypoxemia. *Respiratory research* 2023, 24(1):1.
31. Di Fenza R, Shetty NS, Gianni S, et al. High-Dose Inhaled Nitric Oxide in Acute Hypoxemic Respiratory Failure due to COVID-19: A Multicenter Phase 2 Trial. American journal of respiratory and critical care medicine. 2023.
32. van Zyl AGP, Allwood BW, Koegelenberg CFN, Lalla U, Retief F. The effect of inhaled nitric oxide on shunt fraction in mechanically ventilated patients with COVID-19 pneumonia. African journal of thoracic and critical care medicine. 2023;29(2).
33. Bicakcioglu M, Kalkan S, Duzenci D, Yalcinsoy M, Dogan Z, Ozer AB. Inhaled nitric oxide as rescue therapy in severe ARDS cases due to COVID-19 pneumonia: a single center experience. Eur Rev Med Pharmacol Sci. 2023;27(13):6422-6428.
34. Mekontso Dessap A, Papazian L, Schaller M, et al. Inhaled nitric oxide in patients with acute respiratory distress syndrome caused by COVID-19: treatment modalities, clinical response, and outcomes. Annals of intensive care. 2023;13(1):57.
35. Blot PL, C DER, Deniau B, et al. Efficacy of almitrine as a rescue therapy for refractory hypoxemia in COVID and non-COVID acute respiratory distress syndrome. A retrospective monocenter study. Minerva anestesiologica. 2023;89(3):157-165.
